# Molecular encoding of stimulus features in a single sensory neuron type enables neuronal and behavioral plasticity

**DOI:** 10.1101/2023.01.22.525070

**Published:** 2023-01-22

**Authors:** Nathan Harris, Samuel Bates, Zihao Zhuang, Matthew Bernstein, Jamie Stonemetz, Tyler Hill, Yanxun V. Yu, John A. Calarco, Piali Sengupta

**Affiliations:** 1 Department of Biology, Brandeis University, Waltham, MA, USA; 2 Department of Neurology, Medical Research Institute, Zhongnan Hospital of Wuhan University, Wuhan University, Wuhan, China; 3 Department of Cell and Systems Biology, University of Toronto, Toronto, ON, Canada; 4 Current address: Neuroscience Graduate Program, University of Southern California, Los Angeles, CA, USA

## Abstract

Neurons modify their transcriptomes in response to an animal’s experience. How specific experiences are transduced to modulate gene expression and precisely tune neuronal functions are not fully defined. Here, we describe the molecular profile of a thermosensory neuron pair in *C. elegans* experiencing different temperature stimuli. We find that distinct salient features of the temperature stimulus including its duration, magnitude of change, and absolute value are encoded in the gene expression program in this single neuron, and identify a novel transmembrane protein and a transcription factor whose specific transcriptional dynamics are essential to drive neuronal, behavioral, and developmental plasticity. Expression changes are driven by broadly expressed activity-dependent transcription factors and corresponding *cis*-regulatory elements that nevertheless direct neuron- and stimulus-specific gene expression programs. Our results indicate that coupling of defined stimulus characteristics to the gene regulatory logic in individual specialized neuron types can customize neuronal properties to drive precise behavioral adaptation.

## Introduction

Animals must modulate their physiology and behavior appropriately in response to changing environmental conditions. A major driving force underlying long-term plasticity is the ability of cells to reformat the usage of their genomes as a function of their experience (Lee et al., 2008; Roselli et al., 2021; Bliim et al., 2016; Yap and Greenberg 2018). In the nervous system, different patterns of activity are translated into unique gene expression programs across neuronal ensembles comprised of multiple neuronal subtypes (Spiegel et al., 2014; Hu et al., 2017; Tyssowski et al., 2018; Hrvatin et al., 2018; Lacar et al., 2016; Wu et al., 2017). However, since neurons exhibit extensive heterogeneity in their properties even within a defined subtype (Peng et al., 2021; Zeng, 2022; Scala et al., 2021; Cembrowski and Spruston, 2019), whether behavioral plasticity in response to a defined experience arises due to gene expression changes across neuronal populations or in a circumscribed neuronal subset is largely unknown. Moreover, it is challenging to establish how changes in the expression of individual genes influence neuronal function and behavior.

Sensory neurons in the periphery are typically tuned to defined stimulus parameters and also exhibit experience-dependent response plasticity. While sensory neurons rapidly adapt to persistent sensory cues via non-transcriptional mechanisms (Martelli and Storace, 2021; Pugh et al., 1999), sensory neurons are now known to also alter their molecular profiles in response to long-term environmental experiences. Circadian light-dark cycles alter phototransduction genes in mammalian photoreceptors; these expression changes may underlie circadian modulation of visual processing (McMahon et al., 2014;Hölter et al., 2012; Storch et al., 2007; Korenbrot and Fernald, 1989). In recent work, it has been shown that the gene expression profiles of olfactory receptor neurons in the mouse olfactory epithelium reflect the animal’s past odor experience, and in turn predict the animal’s response to incoming chemical stimuli (Tsukahara et al., 2021). Whether specific stimulus features are encoded in expression changes of defined sensory gene subsets, and the causal relationships between these changes and behavioral plasticity have yet to be definitively described. It is also currently unclear whether canonical activity-dependent gene regulatory pathways described in central neurons (Flavell and Greenberg, 2008; West et al., 2001) generalize to sensory neurons to modulate their unique response profiles.

*C. elegans* sensory neurons provide an excellent experimental system in which to systematically describe gene expression changes in response to precisely controlled sensory stimuli, and to causally and mechanistically link these expression changes to behavioral plasticity. Sensory neuron types in *C. elegans* are typically comprised of a single left/right neuron pair that are genetically accessible at single neuron resolution (White et al., 1986; Taylor et al., 2021). The sensory responses of many of these neurons have been extensively characterized, and behaviors driven by these neurons are known (Bargmann, 2006; Goodman and Sengupta, 2019). Experience-dependent changes in the expression of a limited number of sensory neuron genes, and the roles of these expression changes in driving behavioral plasticity have been previously described in *C. elegans* (Ryan et al., 2014, Meisel et al., 2014; Gruner et al., 2014; Peckol et al., 2001; Sims et al., 2016; Kaye et al., 2009), suggesting that sensory stimulus-driven activity may globally modulate gene expression programs in individual sensory neuron subtypes to alter behavioral outputs in this organism.

Temperature plays a particularly critical role in regulating both physiology and behavior of *C. elegans* (Hedgecock and Russell, 1975; Ailion and Thomas, 2000; Klass, 1977; Golden and Riddle, 1984a). These nematodes are responsive to multiple aspects of their temperature experience including their actual growth temperature as well as temperature changes. Growth temperature regulates lifespan and a developmental decision between alternate larval life-cycles (Klass, 1977; Ailion and Thomas, 2000; Golden and Riddle, 1984a), and adult animals exhibit robust experience-dependent navigation behaviors in response to temperature changes on spatial thermal gradients (Hedgecock and Russell, 1975). Temperature information is largely transduced by the single AFD thermosensory neuron pair to modulate both development and behavior (Goodman and Sengupta, 2019). We and others previously showed that past and current temperature experience alters the expression of a small number of genes in AFD (Yu et al., 2014; Chen et al., 2016; Kobayashi et al., 2016; Servello et al., 2022), raising the possibility that encoding of different features of the temperature stimulus in the gene expression profile of AFD may underlie the ability of AFD to modulate diverse aspects of the organism’s physiology.

Here we use single neuron type profiling to show that the gene expression program in AFD can reflect multiple aspects of the animal’s temperature experience including the duration, as well as the magnitude of temperature change and its absolute value. We show that the gene expression programs in AFD can encode the absolute growth temperature as well as the magnitude and duration of experienced temperature change, and we causally link expression changes in specific genes to plasticity in AFD-driven functions. We find that expression levels of the conserved DAC-1 Dachsund/DACH1 transcription factor report absolute warm growth temperatures to modulate a larval developmental decision. In contrast, expression changes in an AFD-specific transmembrane protein C12D8.15 (henceforth referred to as PYT-1; see below) report a rapid large magnitude temperature change, and this expression change is essential to drive AFD-mediated thermotaxis behavioral plasticity specifically under these conditions. Temperature alters gene expression in AFD in part via the CRH-1 cAMP response element binding protein (CREB), and we demonstrate that deletion of a single cAMP response element (CRE) motif in the endogenous *pyt-1* promoter is sufficient to abolish temperature-dependent expression changes and neuronal plasticity. Together, these findings describe the mechanistic trajectory from a precisely defined sensory stimulus to neuronal gene expression plasticity, and establish the consequences of these expression changes on organismal and behavioral adaptation at single gene and neuron resolution *in vivo*.

## Results

### Identification of temperature experience-regulated genes in AFD via TRAP-Seq

**Fig. 1.**
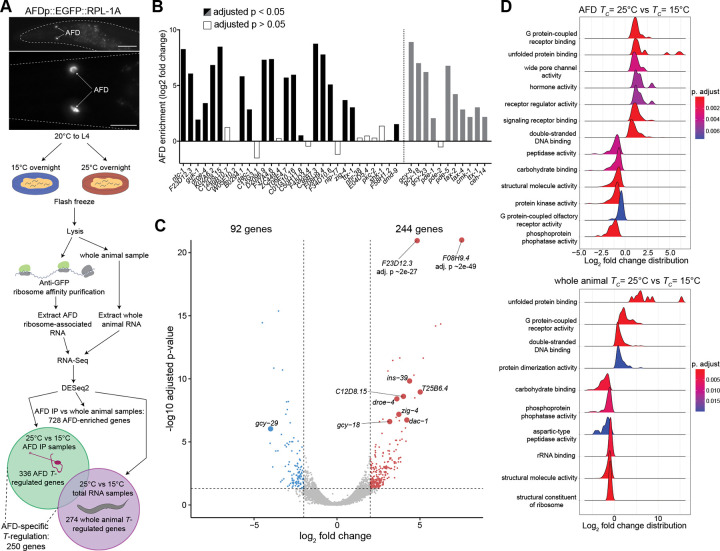
TRAP-Seq profiling of AFD identifies temperature experience-regulated genes. A) (Top) Representative images of the EGFP-tagged RPL-1A ribosomal subunit expressed in AFD in the strain used for TRAP-Seq. (Bottom) TRAP-Seq pipeline to identify temperature-regulated genes in AFD and/or whole animals. IP – immunoprecipation. Scale bars: (top) 50 μm, (bottom) 20 μm; anterior at left. B) Quantification of enrichment in AFD IP vs whole animal samples for the top 30 AFD-enriched genes in the CeNGEN gene expression database (Taylor et al., 2021) (black bars), and for AFD-expressed genes previously implicated in AFD functions (grey bars). Adjusted p-values were calculated by a Wald test performed by DESeq2 (Love et al., 2014). C) Volcano plot of differential gene expression in AFD from animals cultivated at 25°C vs 15°C overnight. Vertical and horizontal dashed lines indicate gene expression changes that are log2 fold change > 2 or < −2 and significant at adjusted p-value < 0.05, respectively. Red and blue dots indicate genes exhibiting significantly higher expression at 25°C or 15°C, respectively. Genes further examined in this work are shown with larger dot sizes. D) Gene set enrichment analysis of temperature-regulated genes in AFD (top) and whole animals (bottom). gseaGO from the clusterProfiler R package (Yu et al., 2012) was used to find enriched gene sets associated with “molecular function” ontology terms. Gene sets with redundant ontology terms were removed manually. Also see Figure S1.

To assess the temperature-regulated gene expression program in AFD, we identified AFD-enriched mRNAs in animals grown at different temperatures using translating ribosome affinity purification (TRAP) (Heiman et al., 2008; Gracida and Calarco, 2017). TRAP permits flash freezing of animals (see Methods) allowing for more precise measurements of temperature-regulated expression changes as compared to experimental approaches such as cell sorting that require exposure to different temperatures for prolonged time periods (Taylor et al., 2021; Kaletsky et al., 2016). We generated transgenic animals stably expressing the GFP-tagged RPL-1A large ribosomal subunit exclusively in AFD (Figure 1A), and deep sequenced isolated ribosome-associated mRNAs from AFD in adult animals, as well as RNA obtained from whole animal lysates, from cultures grown overnight (~16 hrs) at either cold (15°C) or warm (25°C) temperatures (Figure 1A; see Methods).

Principal component analysis indicated that the mRNA profiles largely clustered based on tissue (AFD vs whole animal) and temperature experience (15°C vs 25°C) (Figure S1A). Comparison of gene expression in AFD versus whole animal samples identified 728 AFD-enriched genes independent of temperature regulation (Figure 1A). Comparing the set of AFD-enriched genes detected by our TRAP experiments with those identified by the CeNGEN *C. elegans* single gene expression resource (Taylor et al., 2021) showed substantial overlap (Figure 1B). Additionally, previously identified AFD-expressed genes required for AFD development and/or function were also significantly enriched in the AFD-specific TRAP dataset (Figure 1B). Together, these results indicate that we successfully identified AFD-specific genes via TRAP.

We next compared the gene expression profiles of AFD in animals grown at cold and warm temperatures. The expression of a total of 336 genes was altered by temperature experience in AFD, with 250 of these genes exhibiting temperature-regulated expression changes that are likely to be AFD-specific (Figure 1A; Supplementary Data). Of the set of 336 genes, mRNA levels of 244 and 92 genes were present at higher levels in animals grown at warm versus cold temperatures, respectively (Figure 1C). Gene Set Enrichment Analysis (Yu et al., 2012) indicated that temperature experience altered expression of genes implicated in stress response pathways in both AFD and whole animal samples, but specifically altered genes predicted to affect neuronal signaling pathways in AFD (Figure 1D, Figure S1B). Moreover, while neuropeptide and guanylyl cyclase gene classes, including the previously characterized AFD-specific temperature-regulated *gcy-18* thermoreceptor molecule (Yu et al., 2014; Takeishi et al., 2016; Inada et al., 2006), were among genes differentially expressed in AFD (Figure S1B,C), no neuronal gene class was enriched among differentially expressed genes in whole animal samples (Figure S1C). Together, these observations indicate that the expression of a subset of genes implicated in neuronal functions is bidirectionally regulated by temperature experience in the AFD thermosensory neurons.

### Temperature regulates gene expression in AFD on multiple timescales

**Fig. 2.**
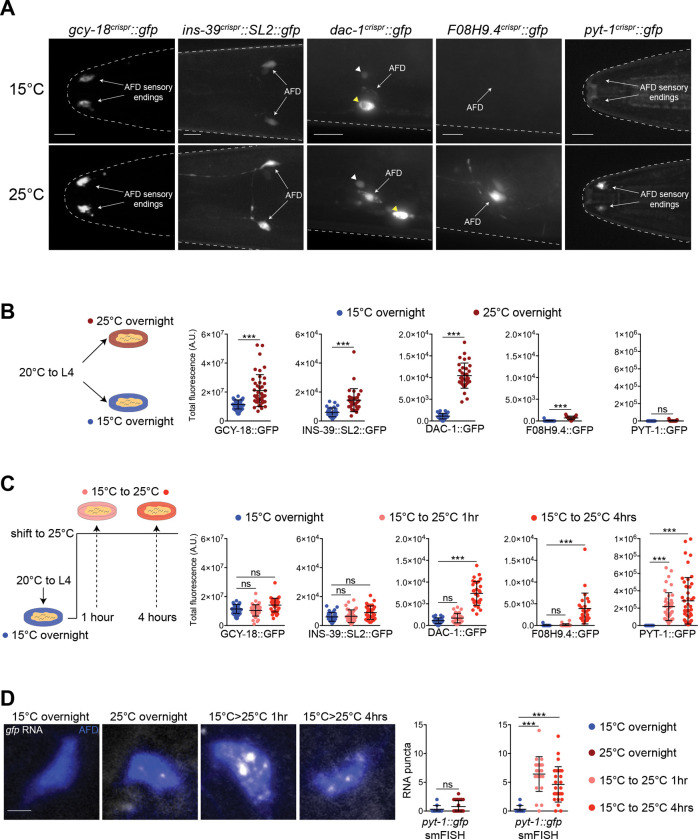
Gene expression in AFD is regulated by the duration of temperature experience. A) Representative images showing expression of indicated endogenously GFP-tagged fusion proteins in the heads of adult hermaphrodites. Animals were grown until the L4 stage at 20°C, shifted to 15°C overnight, and then shifted to 25°C either overnight [*ins-39::SL2::gfp(oy167)*, *gcy-18::gfp(oy165)*] or for 4 hrs [*F08H9.4::gfp(syb5551)*, *pyt-1::gfp(oy169)*, *dac-1::gfp(oy172)*]. AFD soma and sensory endings are indicated. *dac-1::gfp* expression in a hypodermal cell and neuron is indicated by a yellow and white arrowhead, respectively. The worm head is outlined with a dotted line. Anterior is at left. Scale bars: 10 μm. B,C) (Left) Cartoons showing temperature exposure protocols. (Right) Quantification of GFP levels of shown fusion proteins in adult animals grown at the indicated conditions. Each dot represents a measurement from a single AFD neuron. Horizontal and vertical lines indicate mean and SD, respectively. n = 22–48 neurons from at least two biologically independent experiments. For each reporter, measurements for conditions shown in B and C were performed together in one imaging session, and the 15°C overnight control data are repeated in these panels. *** indicates different at p<0.001 (one-way ANOVA with Dunnett’s multiple comparisons correction – GCY-18::GFP, INS-39::SL2::GFP, and DAC-1::GFP, or Kruskal-Wallis test with Dunn’s multiple comparisons correction – F08H9.4::GFP and PYT-1::GFP); ns – not significant. D) (Left) Representative images showing smFISH labeling of *pyt-1::gfp* RNA molecules in AFD neurons of L4 animals. Animals were grown under conditions shown in B and C. Scale bar: 2 μm. (Right) Quantification of *pyt-1::gfp* RNA puncta number in AFD. Horizontal and vertical lines indicate mean and SD, respectively. n = 10–27 neurons. *** indicates different at p<0.001 (Kruskal-Wallis test with Dunn’s multiple comparisons correction); ns – not significant. Also see Figure S2.

To validate and further assess temperature-regulated gene expression patterns in AFD, we examined the expression of a subset of identified temperature-regulated genes using fluorescent reporter-tagged genes. Selected genes are predicted to encode proteins that have already been shown, or are hypothesized, to be involved in regulating AFD thermosensory functions. These include genes encoding receptor guanylyl cyclases (*gcy-18*, *gcy-29*) (Inada et al., 2006; Takeishi et al., 2016), the ortholog of the human DACH1/2 Dachsund transcription factor (*dac-1*) (Colosimo et al., 2004), an insulin-like neuropeptide (*ins-39*) (Servello et al., 2022), a crystallin chaperone protein homologue (*F08H9.4*), an immunoglobulin domain protein (*zig-4*) (Aurelio et al., 2002), a predicted novel transmembrane protein (*pyt-1* – PY domain transmembrane protein, see below), and uncharacterized proteins (*F23D12.3*, *T25B6.4*, and *droe-4*). Genes were tagged with reporter sequences at their endogenous loci via gene editing or expressed from multicopy extrachromosomal arrays as transcriptional reporter gene fusions (Table S1; Figure S2A). All five endogenously tagged genes, and three of five transcriptional reporter gene fusions exhibited expression exclusively in AFD or in a subset of additional cell types at one or both temperatures (Figure 2A, Figure S2B,C, Table S1). Reporter-tagged proteins were localized to the AFD soma/ nuclei, at the AFD sensory endings, or throughout the cytoplasm (Figure 2A). All examined endogenously tagged genes showed increased expression in AFD upon overnight temperature upshifts used for TRAP-Seq experiments with the exception of *pyt-1::gfp(oy169)* (henceforth referred to as *pyt-1::gfp)* (Figure 2A,B). *dac-1::gfp(oy172)* (henceforth referred to as *dac-1::gfp*) is expressed in AFD as well as in a hypodermal cell and an unidentified neuron type (Figure 2A). Levels of this protein were also increased in these cells upon overnight temperature upshift but to a more modest extent (Figure S2D), indicating that temperature-dependent upregulation of this gene is not restricted to AFD under these conditions.

Experience and/or neuronal activity-dependent gene expression changes occur in distinct and partly overlapping temporal waves (Tyssowski et al., 2018; Fowler et al., 2011). To determine whether AFD-expressed genes display distinct underlying temporal expression dynamics, we next examined the expression of reporter-tagged genes in young adult animals after shifting between cold and warm temperatures for different periods of time. Expression patterns could be categorized broadly into three gene groups based on their temporal patterns of expression changes in AFD following the temperature upshift. The first group included *gcy-18* and *ins-39* whose expression levels in AFD were unaltered even upon a 4 hr shift from 15°C to 25°C (Figure 2C). The second group included *dac-1* and *F08H9.4* which exhibited increased expression in AFD at 4 hr after the temperature upshift but not at earlier time points (Figure 2C). We noted that in contrast to the increased expression of DAC-1::GFP in other cell types observed upon overnight exposure to 25°C, levels of this protein were not altered in these cell types at either 1 hr or 4 hr following temperature upshift (Figure S2D). The third group consisted of *pyt-1* whose expression was significantly increased following just 1 hr exposure to 25°C; levels were not further increased with a 4 hr incubation at the warmer temperature (Figure 2C). We infer that the expression levels of a subset of genes in AFD appear to be regulated by the duration of exposure to a new temperature, and that temperature experience regulates gene expression in AFD on multiple timescales and likely via multiple mechanisms.

We next tested whether the observed expression changes arise from transcriptional and/or post-transcriptional changes. We previously showed that upregulation of *gcy-18* expression upon a temperature upshift is primarily mediated via transcriptional mechanisms (Yu et al., 2014). Using smFISH to detect RNAs in AFD, we found that *pyt-1::gfp* RNA was undetectable after cultivation at 15°C in nearly all animals but was strongly upregulated after a 1 or 4 hr shift to 25°C (Figure 2D) recapitulating the expression changes exhibited by the fusion protein. After overnight cultivation at 25°C, *pyt-1::gfp* RNA expression was low but detectable in a subset of animals, although these levels were not significantly different from those in animals cultivated at 15°C (Figure 2D). *pyt-1* may have been identified as an upregulated gene in AFD via TRAP-Seq due to inflation of the fold-change as a consequence of near complete absence of *pyt-1* expression upon cultivation at 15°C. We conclude that warm temperature experience upregulates the expression of a subset of genes in AFD primarily via transcriptional mechanisms.

### Gene expression changes in AFD can reflect the magnitude of experienced temperature change and absolute growth temperature

**Fig. 3.**
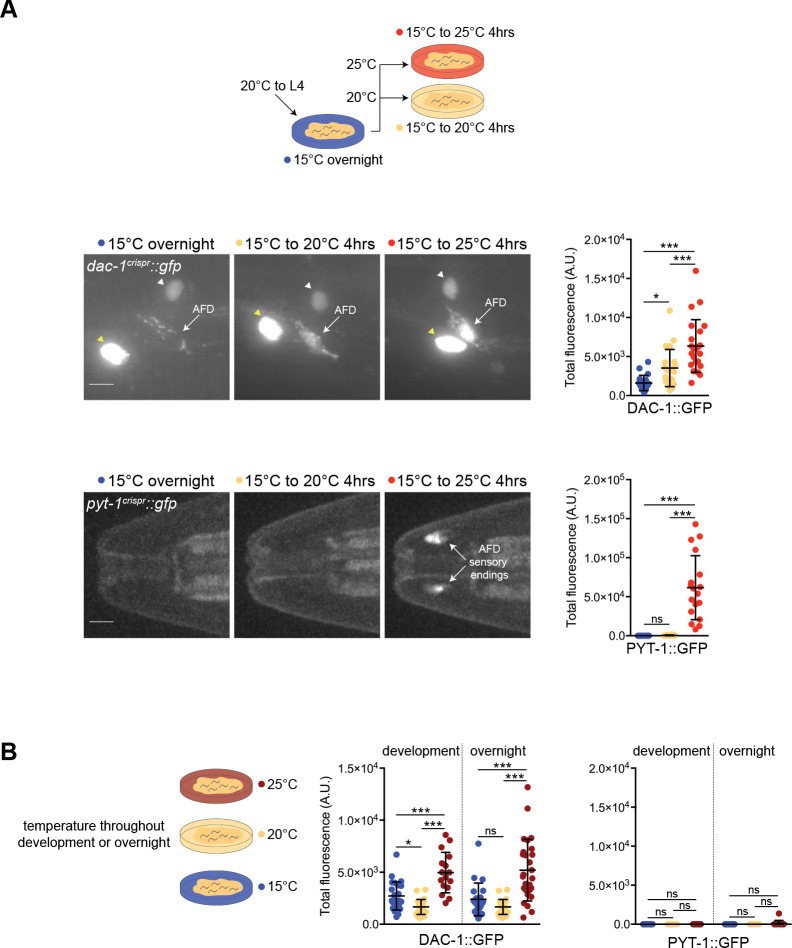
The absolute growth temperature and extent of experienced temperature change are differentially encoded in gene expression patterns in AFD. A) (Top) Cartoon showing temperature exposure protocol. (Middle and bottom) Representative images and quantification of DAC-1::GFP (middle) and PYT-1::GFP (bottom) levels in AFD neurons of adult animals grown at the shown conditions. Yellow and white arrowheads indicate hypodermal and an unidentified neuron expressing DAC-1::GFP, respectively. Scale bars: 10 μm; anterior is at left. Each dot represents a measurement from a single AFD neuron. n = 12–23 neurons from at least two biologically independent experiments. B) (Left) Cartoon showing temperature exposure protocol. (Middle and right) Quantification of DAC-1::GFP and PYT-1::GFP levels in AFD in adult animals grown at the shown conditions. Each dot represents a measurement from a single AFD neuron. Horizontal and vertical lines indicate mean and SD, respectively. n = 10–27 neurons from at least two biologically independent experiments. 20°C measurements are repeated in each reporter expression plot. For all panels, * and *** indicate different at p<0.05 and 0.001, respectively (one-way ANOVA with Dunnett’s multiple comparisons correction – DAC-1::GFP, or Kruskal-Wallis test with Dunn’s multiple comparisons correction – PYT-1::GFP); ns – not significant.

Gene expression changes across neuronal populations can encode specific features of inducing stimuli, including their magnitude and duration (Tyssowski et al., 2018; Beagan et al., 2020; Fields et al., 1997). Since the AFD neuron pair regulates behavioral and physiological outputs in response to both absolute growth temperature and temperature change (Ailion and Thomas, 2000; Clark et al., 2006; Yu et al., 2014; Servello et al., 2022), we tested whether gene expression programs in this single neuron type reflect multiple features of the temperature stimulus. We focused on *dac-1* and *pyt-1* since each exhibited large expression changes but with markedly distinct temporal dynamics after an experienced temperature shift (Figure 2B,C). To first address whether changes in expression of these genes reflect the magnitude of the temperature change, we shifted animals to a new temperature that was 5°C or 10°C warmer than their initial cultivation temperature for 4 hrs (Figure 3A). We found that DAC-1::GFP levels in AFD increased in proportion to the magnitude of the temperature change (Figure 3A). In contrast, levels of PYT-1::GFP were unaltered with a 5°C temperature shift but showed robust upregulation upon a 10°C temperature shift (Figure 3A). We infer that *dac-1* expression is proportional to the magnitude of temperature change, whereas *pyt-1* is instead only upregulated above a large magnitude temperature change threshold, suggesting analog and digital modes of regulation, respectively.

To next test whether levels of these genes can also report the absolute growth temperature as opposed to temperature change, we grew animals at a specific temperature overnight or throughout postembryonic development. DAC-1::GFP levels were low in animals grown under either condition at either 15°C and 20°C, but were consistently elevated in animals grown at 25°C (Figure 3B). Thus, in adult animals that have experienced no change in temperature for a prolonged period of >16 hrs or since hatching, DAC-1 levels are nevertheless elevated in animals cultivated at warm temperatures. In contrast to DAC-1, PYT-1::GFP levels were undetectable in animals grown overnight or throughout postembryonic development at any examined temperature (Figure 3B). We conclude that while levels of DAC-1 can encode both relative temperature change on shorter timescales as well as absolute warm temperature upon prolonged exposure, PYT-1 expression levels only reflect high magnitude relative temperature changes. These observations suggest that different features of the temperature stimulus can be encoded in the gene expression program in AFD, and may enable diverse functional adaptations of AFD to experienced temperature stimuli.

### Temperature experience differentially regulates gene expression in AFD via calcium signaling pathways and a CREB transcription factor

**Fig. 4.**
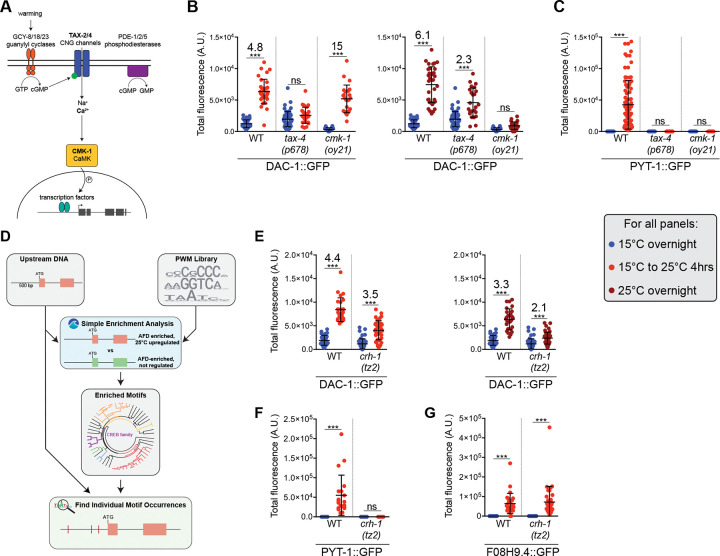
Temperature stimulus features are transduced differentially by calcium signaling pathways and CREB to regulate gene expression. A) Cartoon of the AFD thermosensory signal transduction pathway. B,C,E,F) Quantification of DAC-1::GFP (B,E) and PYT-1::GFP (C,F) levels in adult animals grown at the indicated conditions in tax-4 and cmk-1 (B,C), and crh-1 (E,F) mutants. All reporters were tagged endogenously with GFP. Each dot is a measurement from a single AFD neuron. Horizontal and vertical lines indicate mean and SD, respectively. n = 9–85 neurons from at least two biologically independent experiments. A subset of WT data for DAC-1::GFP measurements is repeated from Figure 2B,C. *** indicates different at p<0.001 (one-way ANOVA with Dunnett’s multiple comparisons correction – DAC-1::GFP, Mann-Whitney test – PYT-1::GFP); ns – not significant. Numbers at top indicate fold-change between indicated conditions. D) Schematic of the analysis pipeline used to predict transcription factor binding sites upstream of temperature-regulated genes (see Methods). PWM: position weight matrix. G) Quantification of endogenously tagged F08H9.4::GFP levels in adult animals grown at the indicated conditions in *crh-1* mutants. Each dot is a measurement from a single AFD neuron. Horizontal and vertical lines indicate mean and SD, respectively. n = 20–34 neurons from at least two biologically independent experiments. *** indicates different at p<0.001 (Mann-Whitney test).

How are different temperature stimulus characteristics transduced to drive expression changes in AFD? Neuronal depolarization and calcium influx modulate gene expression via signaling pathways that include calcium/calmodulin-dependent protein kinases (West et al., 2001). Similarly, in AFD, we and others previously showed that the temperature-dependent expression changes of a subset of genes are regulated by calcium influx via the TAX-2/TAX-4 cGMP-gated channels and the CMK-1 calcium/calmodulin-dependent protein kinase 1 (Figure 4A) (Satterlee et al., 2004; Yu et al., 2014; Kobayashi et al., 2016; Servello et al., 2022). We tested the extent to which these molecules are also required for translating different aspects of the temperature stimulus into specific gene expression programs in AFD.

Consistent with a requirement for calcium influx upon a temperature change to alter gene expression, upregulation of both PYT-1::GFP as well as DAC-1::GFP upon a temperature upshift was fully dependent on TAX-4 (Figure 4B,C). We also previously showed that elevated expression of *ins39* upon a temperature shift requires TAX-4 (Servello et al., 2022). However, the requirement for CMK-1 was distinct such that only PYT-1::GFP but not DAC-1::GFP expression upon a temperature upshift required CMK-1 (Figure 4B,C). In contrast, a mutation in tax-4 only partially disrupted elevation of DAC-1::GFP levels upon prolonged growth at warm temperatures, although this upregulation was fully dependent on CMK-1 (Figure 4B,C). We conclude that calcium influx through TAX-4 acts via both CMK-1-dependent and -independent pathways to alter gene expression upon a temperature upshift, but that absolute warm temperature information is encoded via activation of CMK-1 by alternate pathways (see Figure S3D for summary).

We next investigated the downstream transcriptional regulatory mechanisms that drive temperature experience-dependent gene expression changes. Restricting our search space to sequences 500 bp upstream of the translation start site of each temperature-upregulated gene in AFD, we first identified matches to a curated list of transcription factor binding motifs identified from analyses in multiple organisms (Figure 4D) (Bailey and Grant, 2021; Weirauch et al., 2014). 57 motifs were specifically enriched in the set of AFD-expressed and temperature-regulated but not temperature-insensitive genes (Figure 4D, see Methods). These motifs were then clustered into 27 groups representative of transcription factor families that bind a similar motif (Figure 4D) (Castro-Mondragon, 2017). Of the motif clusters identified, we noted one set that is predicted to be recognized by transcription factors related to the neuronal activity-dependent transcription factor cyclic AMP response element-binding protein (CREB) (Sheng et al., 1990; Kaang et al., 1993) (Figure 4D). The C. elegans CREB ortholog CRH-1 is expressed in AFD and has previously been implicated in regulating thermosensory behaviors and temperature- and AFD-dependent developmental pathways (Nishida et al., 2011; Nakano et al., 2022; Chen et al., 2016; Park et al., 2021).

We tested whether CRH-1 mediates temperature experience-dependent regulation of a subset of identified AFD-expressed genes. In response to a temperature upshift for 4 hrs, elevated expression of *dac-1* and *pyt-1* was partly or fully CRH-1-dependent, respectively (Figure 4E,F). However, at this timepoint, increased expression of *F08H9.4* was CRH-1-independent (Figure 4G), indicating that factors other than CRH-1 also read out temperature shift information to alter gene expression at this timepoint. CRH-1 also partly abrogated increased DAC-1::GFP levels upon overnight growth at warm temperatures indicating that in addition to mediating temperature change information (Figure 4E), CRH-1 may also integrate warm growth temperature information to regulate gene expression. These results highlight the diversity of regulatory pathways that translate specific aspects of the temperature stimulus into unique gene expression programs in AFD (summarized in Figure S3D).

### CREB acts both directly and indirectly to regulate gene expression in response to different features of the temperature stimulus

**Fig. 5.**
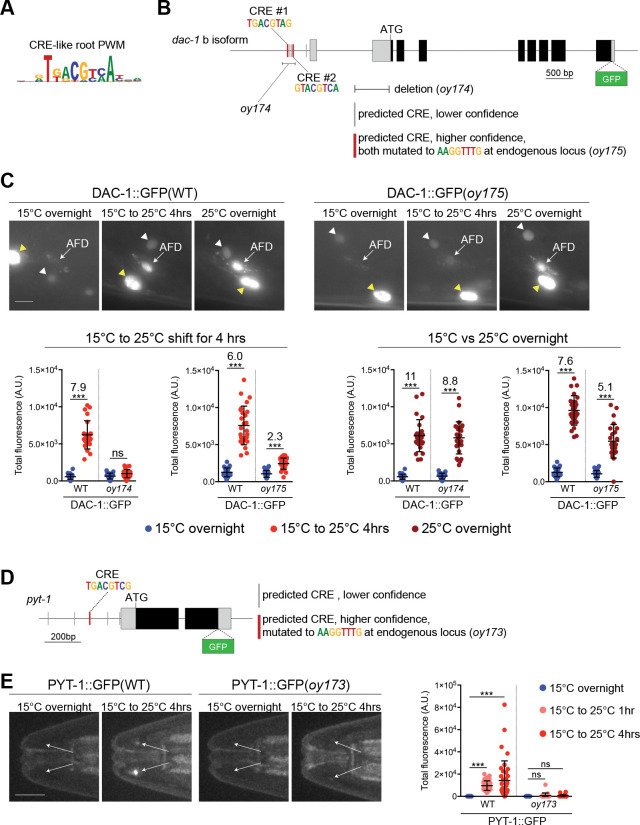
CREB can act directly to regulate gene expression in response to a temperature upshift. A) Root PWM derived from CRE-related PWMs generated with RSAT (Castro-Mondragon et al., 2017). B) Genomic structure of the dac-1b::gfp locus. Predicted CRE motifs within 450 bp upstream of the first exon of *dac1b* are depicted as vertical bars. The extent of the oy174 deletion and mutations in two predicted CREs (oy175) indicated by thick vertical red bars were generated in the endogenous *dac-1::gfp(oy172)* reporter (see Methods). Thin lines, grey boxes, and black boxes indicate upstream sequences/introns, untranslated regions, and coding sequences, respectively. C) (Left) Representative images of endogenously tagged DAC-1::GFP expression under wild-type or mutated (2XCRE mutant; oy175) regulatory sequences in adult animals grown at the indicated conditions. White and yellow arrowheads indicate a hypodermal cell and neuron, respectively. Scale bar: 5 μm. Anterior is at left. (Right) Quantification of endogenously tagged DAC-1::GFP levels in AFD in adult animals expressed under wild-type, or mutated (deleted CREs - oy174; two mutated CREs - oy175). Each dot is a measurement from a single AFD neuron. Horizontal and vertical lines indicate mean and SD, respectively. n = 23–27 neurons from at least two biologically independent experiments. A subset of WT data for DAC-1::GFP measurements is repeated from Figure 2B,C. *** indicates different at p<0.05 and 0.001, respectively (one-way ANOVA with Dunnett’s multiple comparisons correction). ns – not significant. Numbers at top indicate fold-change between indicated conditions. D) Structure of the *pyt-1::gfp* gene locus. Predicted CREs are depicted as vertical bars. The DNA sequence indicated by the thick vertical red bar was mutated in the endogenous *pyt-1::gfp* reporter to generate oy173 (see Methods). Thin lines, grey boxes, and black boxes indicate upstream sequences/introns, untranslated regions, and coding sequences, respectively. E) (Left) Representative images of endogenously tagged PYT-1::GFP expression at the AFD sensory endings (arrows) under wild-type or mutated (CRE mutant; oy173) regulatory sequences in adult animals grown at the indicated conditions. Scale bar: 10 μm. Anterior is at left. (Right) Quantification of endogenously tagged PYT-1::GFP levels expressed under wild-type or mutated (CRE mutant; oy173) regulatory sequences in adult animals grown at the indicated conditions. Each dot is a measurement from a single AFD neuron (arrows). Horizontal and vertical lines indicate mean and SD, respectively. n = 20–34 neurons from at least two biologically independent experiments. *** indicates different at p<0.001 between indicated conditions; ns – not significant (Kruskal-Wallis test with Dunn’s multiple comparisons correction); ns – not significant. Also see Figure S3.

We next tested whether CRH-1 acts directly or indirectly to regulate temperature experience-dependent expression of *dac-1* and *pyt-1* in AFD. The *dac-1* locus is predicted to encode two isoforms (DAC-1A and DAC-1B) that differ at their N-termini (www.wormbase.org). A transcriptional reporter driven under 2.4 kb of regulatory sequences upstream of the dac-1a isoform drove expression only in hypodermal cells, whereas a reporter driven under 450 bp of promoter sequences that include part of the dac-1b 5’UTR and upstream DNA drove expression specifically in AFD (Figure S3A). Moreover, expression of the dac-1bp::gfp reporter was strongly upregulated following a temperature upshift (Figure S3B). Deleting 4 of 5 predicted CREB binding sites (CREs - cAMP response elements; Figure 5A,B) at the endogenous dac-1b promoter locus resulted in significant loss of temperature-regulation of DAC-1::GFP after a 4 hr temperature upshift (Figure 5C). A similar result was obtained upon mutating only two of the highest confidence predicted CREs at the endogenous locus (Figure 5C). However, these CRE mutations had little to no effect on *dac-1::gfp* expression levels in response to absolute warm growth temperatures (Figure 5C). We also identified one high confidence CRE and four additional lower confidence CREs in the upstream 500 bp regulatory sequences of *pyt-1* (Figure 5D). Mutating the high confidence CRE at the endogenous locus resulted in nearly complete loss of temperature-dependent upregulation of *pyt-1* (Figure 5E). This CRE was also conserved in the upstream sequences of *pyt-1* orthologs in a subset of nematode species (Figure S3C). We conclude that CRH-1 directly binds to CREs to regulate both *dac-1* and *pyt-1* expression upon a temperature shift, but that CRH-1 may act indirectly to modulate *dac-1* levels in response to warm growth temperature.

### Warm growth temperature information is integrated in DAC-1 expression levels in AFD to modulate a developmental decision

The differential regulation of genes such as *dac-1* and *pyt-1* in AFD by different temperature stimulus features suggests that these molecules may regulate distinct AFD properties as a function of the animal’s temperature experience. Since DAC-1 levels reflect both a short-term temperature shift as well as absolute warm growth temperatures, we explored the contributions of this protein to shaping AFD-driven processes.

When placed on thermal gradients. C. elegans preferentially moves towards the temperature they experienced in the past 3–4 hrs; this behavioral preference is altered upon exposure to a new temperature for a similar time period (Hedgecock and Russell, 1975). To test whether increased expression of *dac-1* upon a temperature upshift for 4 hrs influences thermotaxis behavioral plasticity, we examined the navigation behavior of *dac-1* animals grown at 15°C and shifted to 25°C for 4 hrs in high resolution thermotaxis behavioral assays. However, *dac-1* mutants exhibited no defects in thermotaxis behavior upon growth at either 15°C or following a temperature upshift to 25°C for 4 hrs (Figure S4A). DAC-1 may regulate AFD-driven behaviors under specific conditions or mediate as yet unknown AFD functions in adult animals experiencing this temperature change.

**Fig. 6.**
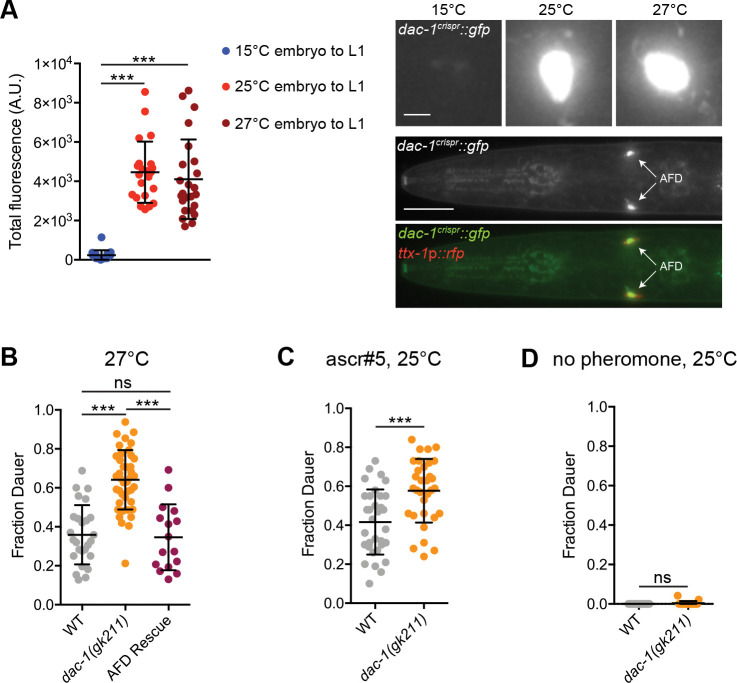
Upregulation of *dac-1* in response to warm growth temperatures regulates the dauer developmental decision. A) (Left) Quantification of DAC-1::GFP levels in AFD in L1 animals grown at the shown conditions. Each dot represents a measurement from a single AFD neuron. Horizontal and vertical lines indicate mean and SD, respectively. n = 19–24 neurons from at least two biologically independent experiments. (Right top) Representative images of DAC-1::GFP expression in AFD of L1 larvae at the indicated temperatures. (Right bottom) Colocalization of DAC-1:GFP with ttx-1p::rfp in AFD soma. Scale bar: (top) 2 μm, (bottom) 100 μm. B-D) Dauers formed by animals of the indicated genotypes. Each dot indicates the fraction of dauers formed in a single assay. Horizontal and vertical lines indicate mean and SD, respectively. n = 16–42 assays from at least two biologically independent experiments. dac-1a(cDNA)::SL2::mCherry was expressed under the gcy-8 promoter in C. For all panels, *** indicates different at p<0.001, (A - one-way ANOVA with Dunnett’s multiple comparisons correction, B - one-way ANOVA with Tukey’s multiple comparisons correction, C,D - t test); ns – not significant. Also see Figure S4.

Warmer growth temperatures (25°C) enhance entry of C. elegans larvae into the stress-resistant dauer developmental stage under defined environmental conditions (Golden and Riddle, 1984a; Golden and Riddle, 1984b; Fielenbach and Antebi, 2008). Thus, growing larvae at 27°C, or at 25°C with added exogenous pheromone preferentially promotes dauer arrest over continued reproductive growth (Ailion and Thomas, 2000; O’Donnell et al., 2018; Golden and Riddle, 1984b; Ailion and Thomas, 2000). As in adult animals, *dac-1* expression was also increased in AFD in L1 larvae grown at warm temperatures (Figure 6A), suggesting that DAC-1 may influence this temperature-regulated developmental decision. We found that a significantly larger fraction of *dac-1* mutant animals entered the dauer stage when grown at 27°C (Figure 6B); this phenotype was similar to those of *ttx-1* mutants in which the AFD neurons exhibit developmental defects (Ailion and Thomas, 2000). The enhanced dauer formation defect of *dac-1* mutants was fully rescued by expression of DAC-1 specifically in AFD (Figure 6B). Similarly, more *dac-1* mutants exhibited dauer entry at 25°C in the presence but not absence of low added concentrations of the ascr5 pheromone (Figure 6C,D). Dauer formation is driven in part via downregulation of TGF-beta and insulin signaling from the ASI and ASJ sensory neurons in the head of C. elegans (Fielenbach and Antebi, 2008; O’Donnell et al., 2018). However, expression of neither *daf-7* TGF-beta nor of the *daf-28* insulin-like peptide gene was significantly altered in *dac-1* L1 larvae (Figure S4B,C), indicating that DAC-1 may regulate other neuroendocrine genes to suppress dauer formation at warm growth temperatures. We infer that DAC-1 levels encode warm growth temperature information in the AFD neurons of larvae to appropriately modulate the dauer developmental decision.

### Direct CREB-mediated regulation of *pyt-1* expression in AFD links the experience of a specific temperature change to thermosensory behavioral plasticity

**Fig. 7.**
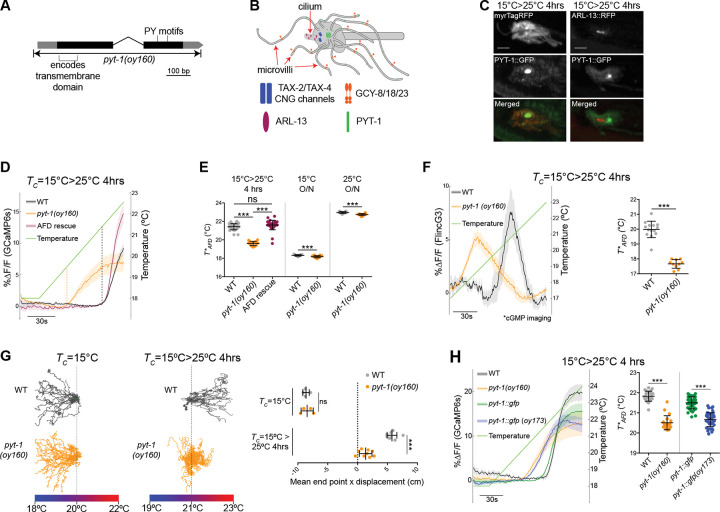
CREB-mediated upregulation of the *pyt-1*-encoded transmembrane protein drives thermosensory behavioral plasticity in response to a specific temperature experience. A) *pyt-1* genomic locus and oy160 deletion boundaries. Black and grey boxes indicate coding DNA and untranslated regions, respectively. Sequences encoding the predicted transmembrane domain and location of the PY motifs are indicated. B) Cartoon of the AFD sensory ending and localization of thermotransduction proteins (Nguyen et al., 2014; this work). C) Representative images of PYT-1::GFP localization within the AFD sensory endings at the indicated temperature conditions. AFD membrane and cilia were visualized with ttx-1p::myrTagRFP and ttx-1p::ARL-13::RFP, respectively. Scale bars: 2 μm. Anterior is at left. D) GCaMP traces acquired from AFD in adult animals grown under the indicated conditions during the shown temperature ramp protocol (green line). Thick lines and shading indicate the average deltaF/F change and SEM, respectively. Dashed vertical lines indicate the corresponding T*AFD for wild-type and *pyt-1(oy160)* animals. The AFD rescue transgene was *gcy-8p::pyt-1(genomic)::SL2::mCherry*. E) Quantification of T*AFD for animals grown under the indicated conditions. Each dot is a measurement from a single animal. Horizontal and vertical lines indicate mean and SD, respectively. n = 12–37 animals from at least two biologically independent experiments. F) (Left) FlincG3 cGMP indicator traces from AFD in adult animals during the shown temperature ramp protocol (green line). Traces are corrected for photobleaching by subtraction of an exponential fit curve for visualization (see Methods). Thick lines and shading indicate the average deltaF/F change and SEM, respectively. (Right) Quantification of T*AFD from traces shown at left. Each dot is a measurement from a single animal. Horizontal and vertical lines indicate mean and SD, respectively. n = 10–14 animals from at least two biologically independent experiments. G) (Left) Representative thermotaxis behavior trajectories of adult wild-type and *pyt-1(oy160)* animals in a single assay on thermal gradients grown at the indicated conditions. Vertical dashed lines indicate the starting temperature on the gradient. (Right) Quantification of thermotaxis assay end points relative to starting position. Each dot indicates the average end point distance of all animals in a single assay. Vertical and horizontal lines indicate mean and SD, respectively. n = 4–9 assays of 15–25 animals each from at least two independent days. H) (Left) GCaMP traces from AFD in adult animals during the temperature ramp protocol (green line). Thick lines and shading indicate the average deltaF/F change and SEM, respectively. (Right) Quantification of T*AFD from traces shown at left. Each dot is a measurement from a single animal. Horizontal and vertical lines indicate mean and SD, respectively. n = 20–44 animals from at least two biologically independent experiments. For all panels, *** indicates different at p<0.001 (E - one-way ANOVA with Tukey’s multiple comparisons correction - 15°C>25°C 4hrs, t test - 15°C O/N and 25°C O/N, F-I - t test,); ns – not significant. Also see Figure S5.

The rapid upregulation of *pyt-1* expression only in response to a large magnitude temperature upshift suggested that this molecule may specifically link transcriptional changes to neuronal and behavioral plasticity in response to experienced temperature change. *pyt-1* encodes a small single transmembrane domain protein (Figure 7A) and is conserved in a subset of both free-living and parasitic nematodes but not in other species (Figure S5A). The predicted C-terminal domain of PYT-1 contains two sequences (PPxY and LPxY, together referred to as a PY motif) (Figure 7A, Figure S5B) which have been shown to bind the conserved Group 1 WW protein-protein interaction motif present in many proteins with a range of functions (Salah et al., 2012; Huang et al., 2020). To begin to explore a function for PYT-1, we first characterized the localization of PYT-1 at the AFD sensory endings. Thermosensory signaling proteins exhibit differential localization within the AFD sensory endings such that the thermosensor receptor guanylyl cyclases including GCY-18 are localized to finger-like microvilli but are excluded from the small cilium, whereas the TAX-2 and TAX-4 thermotransduction channels are restricted to the proximal ciliary region (Figure 7B) (Nguyen et al., 2014). Functional PYT-1::GFP (see Figure 7H) was enriched in a region at the base of the AFD cilium and also present in dimmer puncta distributed throughout the AFD microvilli but was excluded from the cilium itself (Figure 7B,C).

To investigate *pyt-1* function, we generated a null allele of *pyt-1* via gene editing (Figure 7A). The overall morphology of the AFD sensory endings was not significantly altered in *pyt-1* mutants (Figure S5C). We next assessed temperature-driven changes in intracellular calcium dynamics in the AFD sensory endings using the genetically encoded GCaMP6s calcium sensor. In wild type animals, AFD is rapidly activated by warming stimuli above a precise threshold temperature (T*AFD) that is altered in a temperature experience-dependent manner on a fast minutes- and slower timescale of 3–4 hrs (Kimura et al., 2004; Clark et al., 2006; Ramot et al., 2008; Yu et al., 2014). This flexibility in T*AFD together with plasticity in AFD synaptic output drives plasticity in thermotaxis navigation behaviors (Ramot et al., 2008; Hawk et al., 2018; Clark et al., 2006; Kimura et al., 2004; Ohnishi et al., 2011). We found that the T*AFD of *pyt-1* mutants was significantly lower than that of wild-type animals when animals experienced a 15°C to 25°C upshift for 4 hrs (Figure 7D,E), but showed no defects in the fast component of T*AFD adaptation following a temperature upshift for 10 mins (Figure S6C). Moreover, unlike the rapid activation of calcium responses in wild type animals, AFD neurons in *pyt-1* mutants responded to warming with a gradual increase in calcium (Figure 7D). Response phenotypes were fully rescued upon AFD-specific expression of GFP-tagged PYT-1 under a temperature-insensitive promoter (Figure 7E). Remarkably, T*AFD as well as the dynamics of the calcium response in AFD were only altered to a minor extent in *pyt-1* mutants when grown overnight at either 15°C or 25°C when expression levels of this gene are low (Figure 7E, Figure S6A,B). These observations suggest that PYT-1 plays a specific role in tuning AFD thermosensory response plasticity at an hours-long timepoint after a temperature upshift that coincides temporally with upregulated expression of this gene.

AFD responds to warming stimuli by increasing intracellular cGMP levels via the thermoreceptor guanylyl cyclases; cGMP in turn opens the TAX-2 and TAX-4 cGMP-gated channels to allow calcium influx and depolarization (Goodman and Sengupta, 2019). To determine whether PYT-1 functions upstream or downstream of cGMP production in the signal transduction cascade, we next imaged changes in cGMP levels at the AFD sensory endings in response to a warming stimulus using the genetically encoded FlincG3 fluorescent cGMP sensor (Bhargava et al., 2013; Woldemariam et al., 2019). The AFD neurons in *pyt-1* mutants were again activated at a significantly lower temperature compared to wild type following a 4 hr temperature upshift (Figure 7F). Similar to calcium response dynamics, cGMP levels also rose gradually in AFD in *pyt-1* mutants compared to the rapid increase observed in wild-type AFD (Figure 7F), indicating that PYT-1 likely acts at the first step of AFD thermosensory signal transduction upon a temperature upshift for 4 hrs. PY motif containing proteins can be direct targets of protein degradation by the Nedd4 family of E3 ubiquitin ligases via interaction with their WW domains (Rotin et al., 2001; Harty et al., 2000). A subset of small PY-containing transmembrane proteins that otherwise do not share extensive sequence homology have also been shown to act as adapters to recruit these ligases to target proteins that may themselves lack the PY motif (Myat et al., 2002; Hettema et al., 2004; Gorla et al., 2022). However, the levels of the endogenously tagged GCY-18 guanylyl cyclase was unaltered in *pyt-1* mutant animals after a shift from 15°C to 25°C for 4 hrs (Figure S5C), suggesting that PYT- modulates cGMP levels at the AFD thermosensory endings via alternative mechanisms.

Resetting T*AFD as a function of temperature experienced in the past 3–4 hrs is critical for animals to appropriately shift their temperature preference in thermotaxis navigation behaviors (Goodman and Sengupta, 2019). *pyt-1* mutants were markedly defective in their ability to shift their navigation preference towards warmer temperatures (positive thermotaxis) upon a shift from 15°C to 25°C for 4 hrs consistent with defects in their ability to appropriately adapt T*AFD (Figure 7G), but moved similarly to wild-type animals on an isothermal plate at 20°C (Figure S6D). However, reflecting low or absent *pyt-1* expression upon overnight growth at 15°C and no effect on T*AFD in *pyt-1* mutants under these conditions, thermotaxis navigation was unaffected in *pyt-1* animals cultivated at 15°C (negative thermotaxis; Figure 7G).

If temperature-dependent upregulation of *pyt-1* via CREB binding to a single CRE is important for regulating AFD thermosensory plasticity, we reasoned that the *pyt-1* allele in which just the CRE has been mutated (oy173) should exhibit temperature response phenotypes in AFD similar to those of the *pyt-1(oy160)* protein null allele. As shown in Figure 7H, both T*AFD and the dynamics of temperature-evoked intracellular calcium changes in AFD were similarly altered in animals carrying either allele upon a temperature upshift from 15°C to 25°C for 4 hrs. Moreover, similar to *pyt-1* null mutants, AFD temperature responses were largely unaffected in *pyt-1(oy173)* mutants upon overnight growth at either 15°C or 25°C (Figure S6E). crh-1 mutants also exhibited significant defects in T*AFD upon a 15°C to 25°C shift for 4 hrs as well as upon overnight growth at 25°C but a weaker defect upon overnight growth at 15°C (Figure S6F) (Nishida et al., 2011). Together, we conclude that temporally regulated CREB-mediated transcriptional upregulation of *pyt-1* specifically regulates plasticity in AFD thermosensory responses and thermotaxis behavior at a defined timepoint following a large magnitude temperature upshift.

## Discussion

Here we show that specific aspects of an animal’s temperature experience, including the duration and absolute value of the experienced temperature, as well as the extent of temperature change, are quantitatively encoded in the gene expression profile of the single pair of AFD thermosensory neurons in C. elegans. Functional characterization of two genes identified in this work show that their patterns of expression modulation are critical for driving AFD-dependent behavioral and developmental decisions in response to defined aspects of the animal’s temperature experience. We further find that a subset of this experience-dependent regulation is mediated by the CRH-1 CREB transcription factor, and that mutating an individual CREB binding site is sufficient to abolish activity-dependent expression, resulting in defects in neuronal plasticity. Our findings demonstrate encoding of sensory stimulus features in unique gene expression programs thereby coupling stimulus to transcription (Tyssowski and Gray, 2019) in a single sensory neuron type in vivo, and mechanistically describe how these expression changes in single genes influence neuronal and organismal plasticity.

Our results suggest that in AFD, the extent of experienced temperature change is reflected in analog changes in the expression of genes such as *dac-1* thereby providing a mechanism to faithfully report this stimulus feature and modify neuronal function along a continuous axis. Graded CREB-dependent changes in the expression of the *flp-6* neuropeptide gene in AFD at different growth temperatures have previously also been shown to be important for AFD-mediated regulation of longevity at warm temperatures (Chen et al., 2016). Similarly, in rodent olfactory receptor neurons, analog changes in gene expression in response to odor experience history reflect the animal’s odor history and regulate future olfactory responses (Tsukahara et al., 2021). In contrast, a digital regulatory design reports a large magnitude temperature change by upregulating *pyt-1* expression only above a specific stimulus change threshold (Burz et al., 1998; Houchmandzadeh et al., 2002). The magnitude of temperature change has previously been suggested to be proportional to the amplitude of the calcium response in AFD (Clark et al., 2006). Graded changes in second messenger levels could be translated into analog changes in gene expression via non-cooperative binding of transcription factors to multiple cis-regulatory elements (Stewart-Ornstein et al., 2013; Giorgetti et al., 2010). *dac-1* levels also report the absolute warm temperature upon prolonged exposure. Resting calcium levels in AFD have recently been reported to be correlated with growth temperature (Thapliyal et al., 2022; Ippolito et al., 2021). Basal calcium levels in AFD at different temperatures may drive differential expression of downstream targets via distinct regulatory molecules including CMK-1. The ability to couple different features of the thermal stimulus to distinct gene expression programs allows AFD to precisely modulate downstream signaling pathways as a function of the animal’s specific temperature experience.

The critical role of specific gene expression changes in AFD in response to defined stimulus features is exemplified in our functional characterization of both *dac-1* and *pyt-1*. Upregulation of *dac-1* expression in larvae upon prolonged growth at warm temperatures allows animals to integrate their long-term temperature experience to appropriately modulate the dauer decision pathway. Conversely, the rapid and transient upregulation of *pyt-1* following a large magnitude temperature upshift for 1–4 hrs is essential to correctly tune plasticity in AFD thermosensory responses and thermotaxis behavior specifically on this timescale. While it has been suggested that adaptation of only the synaptic output of AFD on an hours-long timescale is sufficient to drive plasticity in thermotaxis behaviors upon a temperature upshift (Hawk et al., 2018), our experiments further confirm that adaptation of the sensory response threshold of AFD on this timescale is also critical for behavioral plasticity (Yu et al., 2014). Homeostatic mechanisms can precisely restore neuronal and/or circuit excitability after activity perturbation by tuning gene expression (O’Leary et al., 2014; Kulik et al., 2019; Tsukahara et al., 2021). Adaptation of the thermosensory response threshold, dependent in part on alteration of expression of genes such as *pyt-1*, allows AFD to correctly establish a new set point following a temperature experience in order to continue to respond bidirectionally with high sensitivity to temperature changes. A feature of many sensory systems is the rapid evolution of sensory receptors that enable animals to adapt to their specialized environmental niche (Bear et al., 2016; Baldwin and Ko, 2020; Yau and Hardy, 2009; Julius and Nathans, 2012). The functioning of sensory systems is further optimized by the presence of species- and modality-specific molecules and mechanisms (Zang and Neuhauss, 2018; Huber, 2001). The transient upregulation of *pyt-1* during a specific phase of AFD response plasticity may be critical for fine-tuning AFD responses possibly via modulation of one or more signaling proteins required for production and/or hydrolysis of cGMP. We propose that species-specific evolution and neuron-specific expression of molecules such as PYT-1, along with acquisition of stimulus-dependent gene regulatory architecture, may provide an elegant mechanism by which sensory transduction mechanisms can be flexibly yet specifically modified.

The roles of regulatory molecules including kinases such as CaMKIV, transcription factors such as CREB and MEF2, as well as subsets of downstream target genes such as Bdnf and Arc in driving experience-dependent neuronal plasticity have largely been studied using gene knockout models (Bourtchuladze et al., 1994; Ernfors et al., 1994; Jones et al., 1994; Shepherd et al., 2006). A confounding factor in these analyses has been the inability to distinguish between possible roles of these molecules in regulating basal vs activity-regulated properties of the expressing neurons. Activity responses can be teased apart from basal expression by identifying and disrupting endogenous activity-dependent cis-regulatory regions, allowing for specific testing of their functional consequences (Hong et al., 2008). The similar response defects of *pyt-1* single CRE mutants and *pyt-1* protein null mutants at 4 hrs after a temperature upshift confirm the critical importance of temperature and CREB-mediated upregulation of this gene in mediating a defined aspect of AFD response plasticity but not other AFD response properties. The ability to specifically target the cis-regulatory site(s) driving activity-dependent gene expression in vivo provides a powerful method to dissect the contributions of individual genes to defined aspects of neuronal and behavioral plasticity.

While the experience-dependent gene expression changes described here are controlled by signaling pathways and transcriptional mechanisms that are common to many neurons, these genes appear to be temperature-regulated only in AFD and play specific roles in regulating AFD properties. Processes that specify a neuron’s static molecular identity may be permissive for their ability to modify that identity in response to encountered stimuli (Hobert and Kratsios, 2019). Thus, specialized activity-dependent gene batteries may be a feature of subsets of neuron types that can only be detected via the use of defined stimuli and characterization of single cell transcriptomes (Yap et al., 2021; Mardinly et al., 2016; Tsukahara et al., 2021; Gray and Spiegel, 2019). A major future challenge will be to comprehensively identify and characterize stimulus-specific gene expression programs at single neuron resolution in vivo, and establish how changes in the expression of single genes and gene batteries encode the animal’s experience to shape defined aspects of its physiological and behavioral responses.

## Methods

### C. elegans strains and genetics

Worms were grown at 20°C on nematode growth media (NGM) plates seeded with E. coli OP50 unless noted otherwise. The wild-type strain used was C. elegans variety Bristol strain N2. Transgenic animals were generated using experimental plasmids at 3–100 ng/ μl and the coinjection marker at 30–50 ng/ μl. Strains containing two or more alleles were generated using standard genetic crosses. The presence of specific molecular lesions and genomic edits was confirmed by sequencing. To stably integrate *ttx-1p::tagRfp* and *ttx-1p::egfp::rpl-1a* sequences into the genome, young transgenic adults carrying extrachromosomal arrays were irradiated with 300 μJ UV light. Animals homozygous for the integrated array were backcrossed six times prior to use. A complete list of all strains used in this work is provided in Supplementary file 1.

### Plasmid construction

Promoter sequences, genomic loci, and cDNAs were amplified from genomic DNA or plasmids, or a C. elegans cDNA library generated from a population of mixed stage animals, respectively (Nechipurenko et al., 2016). Plasmids were constructed using standard restriction enzyme cloning or Gibson assembly (New England BioLabs). All plasmids used in this work are described in Supplementary file 2.

### CRISPR/Cas9-based genome engineering

All crRNAs, tracrRNAs and Cas9 protein were obtained from Integrated DNA technologies (IDT). All gfp insertions were made immediately before the stop codon of each relevant gene in the N2 genetic background.

#### Reporter-tagged alleles: gcy-18(oy165[gcy-18::gfp]:

A donor plasmid was created by first cloning 2 kb genomic DNA surrounding the insertion site into pMC10 (gift of M. Colosimo), followed by addition of AarI restriction sites via site directed mutagenesis (Agilent) to enable insertion of gfp sequences. The gfp sequence was inserted using a crRNA (5’-ctgaatgtagtttgtagtcg-3’) and a gfp donor with 1 kb homology arms amplified from the donor plasmid. *ins-39(oy167[ins-39::SL2::gfp])*: a crRNA (5’-gatggagcattgatcagagc-3’) and a donor containing SL2::gfp amplified using oligonucleotides containing 35 bp homology arms were used to insert SL2::gfp sequences (Servello et al., 2022). *dac-1(oy172[dac-1::gfp])*: A crRNA (5’-gtcttcggacggaaattctg-3’) and a donor containing gfp sequences amplified using oligonucleotides containing 35 bp homology arms were used to insert gfp sequences. F08H9.4(syb5551[F08H9.4::gfp]) was obtained from SunyBiotech. pyt-1(oy169[pyt-1::gfp]): A crRNA (5’-tacatgacgtcatcatatga-3’) and a gfp donor with 400 bp homology arms amplified from a GFP-encoding plasmid were used to insert gfp sequences.

#### Deletion allele:

The *pyt-1(oy160)* deletion allele was generated according to published protocols (Ghanta et al., 2021). 2 crRNAs (5’-tagaaaagtgaattcactgt-3’ and 5’-tggaatgaaacgttgactgc-3’) targeting sequences upstream and downstream of the *pyt-1* coding region and an ssODN donor (5’-cttctaatacgcataaagtcagtatttatagttcagaagtaaatatatttgatttgtgtttagttttttc-3’) containing 35 bp of homology 5’ and 3’ to the cut sites were injected along with Cas9 protein. The injection mix contained: ssODN donor (110 ng/ μl), crRNAs (28 ng/ μl each), tracrRNA (100 ng/ μl), Cas9 (250 ng/ μl), and co-injection marker (myo-3p::mCherry (50 ng/ μl)). The progeny of transgenic animals was subsequently examined for the presence of the desired deletion via sequencing.

#### Mutations in predicted CREs:

To mutate a predicted CRE upstream of *pyt-1(oy169)*, a crRNA (5’-tttgtgacgtcgtctgcaaa-3’) and an ssODN donor (5’-ttttcttctcagtacttgagccaataaaccttttgaaggtttgtctgcaaatccataccaaatctgccaccacaaca-3’) were used to generate *pyt-1(oy169 oy173)*. To mutate CREs upstream of *dac-1(oy172)*, two crRNAs (5’-gatatttttcccagaaagct-3’ and 5’-tctattagatgacgctcatg-3’) and an amplified donor containing mutations in two predicted CREs (tgacgtag>aaggtttg and gtacgtca>aaggtttg) were used to replace the sequences between the crRNA-targeted cut sites and generate *dac-1(oy172 oy175)*. In the process of generating *dac-(oy172 oy175)*, animals carrying a deletion between these two cut sites was also isolated, yielding the allele *dac-1(oy172 oy174)*.

### Translating Ribosome Affinity Purification (TRAP)

TRAP was performed according to published protocols (Gracida and Calarco, 2017) with the following modifications. Worms were cultured on 10X concentrated E. coli HB101 seeded at 2 ml per 15 cm plate. Each plate contained 50,000 growth-synchronized 1-day old adult animals. Animals were growth synchronized by bleaching adults, collecting eggs, and then arresting hatched L1s in the absence of food for 16 hours. A total of 10 plates was used per sample. Worms were cultivated at 20°C until the L4 stage when animals from the same population were placed on plates at 15°C or 25°C overnight. Worm collections were completed within 15 mins (from incubator to flash freezing) to minimize temperature variations. Each immunoprecipitation used worm lysate containing 2000–3000 μg of total RNA per sample. As validation for enrichment of AFD RNA by immunoprecipitation, gcy-8 mRNA was detected using a OneStep RT-PCR kit (QIAGEN) from 3 ng of template RNA. All TRAP experimental steps were performed on samples grown at the two temperatures in parallel using the same TRAP affinity matrix.

### RNA-Sequencing

10 ng RNA from each worm sample was used as input for cDNA synthesis using a SMART-Seq v4 Ultra Low Input RNA kit (Takara). Sequencing libraries were prepared from 500 pg of cDNA with a Nextera XT DNA Library Prep kit (Illumina). Libraries were paired-end sequenced at 75×75 bases on a NextSeq 500 system (Illumina). Sequencing reads were adapter trimmed using cutadapt (Martin, 2011) with the following options: quality cutoff 20, –trim-n, –minimum-length=50, and then mapped to the C. elegans genome (WBcel235/ce11), and counted using STAR (Dobin et al., 2013) with –quantMode GeneCounts. Differential expression analysis was performed in R with DESEQ2 (Love et al,. 2014) (GEO accession number GSE222226). The volcano plot was generated in R using EnhancedVolcano (https://github.com/kevinblighe/EnhancedVolcano). Gene set enrichment analyses were performed in R using the clusterProfiler package (Yu et al., 2012).

### Thermotaxis behavior

Thermotaxis behaviors were performed as previously described (Luo et al., 2014) with the following modifications. Well-fed animals were grown at 20°C to the L4 stage and then cultured at 15°C overnight prior to performing negative thermotaxis assays. For positive thermotaxis assays, animals cultured at 15°C were shifted to 25°C for four hours prior to the assay. The temperature gradient in the assay arena was either 18–22°C (for negative thermotaxis) or 19–23°C (for positive thermotaxis) at a steepness of 0.18°C/cm (Ji et al., 2021). Temperature was controlled by a Peltier system [colder side; H-bridge amplifier (Accuthermo FTX700D), PID controller (Accuthermo FTC100D), Peltier (DigiKey)] and heater system [warmer side; PID controller (Omega CNi3244), solid-state relay Omega SSRL240DC25), and cartridge heaters (McMaster-Carr 3618K403)]. A 22.5 cm square NGM agar pad was used for the assay, and the temperature of agar edges and center were confirmed with a digital thermometer (Fluke Electronics) prior to each assay. 15–25 worms in M9 buffer were placed at the center of the gradient at the start of the assay. Animal movement was imaged at 2 fps for 60 min using a Mightex camera (BTE-5050-U). Animal trajectories were detected and analyzed using custom LabView (National Instruments) and MATLAB (Mathworks) scripts (Gershow et al., 2012) (https://github.com/samuellab/MAGATAnalyzer). All assays were performed using one day-old adults.

### Calcium imaging

Temperature-evoked calcium dynamics in AFD were measured essentially as described previously (Takeishi et al., 2020; Takeishi et al., 2016; Yu et al., 2014) with the following modifications. Animals were cultivated at 20°C until the L4 stage and then shifted to the indicated temperatures. One day-old well-fed adults were immobilized in 10 mM tetramisole on an agarose pad (5% in M9 buffer) on a cover glass, and mounted under a second cover glass for imaging. The sample was transferred to a Peltier temperature control system on the microscope stage. Animals were subjected to linear temperature ramps rising at 0.05°C/s via temperature-regulated feedback using a temperature controller (Accuthermo FTC200), an H-bridge amplifier (Accuthermo FTX700D), and a thermistor (McShane TR91–170). Videos of calcium dynamics at the AFD sensory endings were captured using a Zeiss 40X air objective (NA 0.9) or a Zeiss 10X air objective (NA 0.3) on a Zeiss Axioskop2 Plus microscope, using a Hamamatsu Orca digital camera (Hamamatsu), and MetaMorph software (Molecular Devices). Data were analyzed using custom scripts in MATLAB (Mathworks) (https://github.com/wyartlab/Cantaut-Belarif-et-al.−2020) (Sternberg et al,. 2018; Cantaut-Belarif, 2020). T*AFD was calculated as described previously (Takeishi et al., 2016).

### cGMP imaging

An AFD-expressed FlincG3 cGMP sensor (Woldemariam et al., 2019) was used to measure changes in cGMP concentration. cGMP imaging was performed essentially as described above for calcium imaging. FlincG3 fluorescence was observed to decrease substantially during imaging due to photobleaching. For visualization, the deltaF/F traces were fit with an exponential curve which was subtracted to generate the data in Figure 7F.

### Analyses of GFP fluorescence

To visualize and quantify GFP fluorescence in animals expressing reporter-tagged alleles, well-fed one day-old adult animals grown under indicated temperature conditions were immobilized with 20 mM tetramisole, mounted on 10% agarose pads on slides, and imaged either on a Zeiss Axio Imager M2 epifluorescent microscope with a 63x oil objective (NA 1.4) or on a Zeiss Axio Observer with a Yokogawa CSU-X1 spinning disk confocal head (3i Marianas system) with a 63x oil objective (NA 1.4). AFD was typically identified by co-expression of the AFD-specific marker ttx-1p::tagRfp (Satterlee et al., 2001). Images for quantification were acquired with no red marker in the background. Images were processed in ImageJ, and expression was quantified from a maximum projected z-stack as corrected total cell fluorescence (CTCF) using the equation CTCF = Integrated Density – (Area of selected cell ROI × Mean fluorescence of a nearby background ROI). Protein localization/co-localization at AFD sensory endings was imaged using a Zeiss LSM880 AiryScan Fast Confocal System in the AiryScan configuration with a 63x oil objective (NA 1.4). Animals expressing daf-7p::gfp (ksIs2) and daf-28p::gfp (mgIs40) were cultivated at 27°C under the same conditions used for performing dauer assays (see below) and imaged as L1s 20–24 hrs after egg laying. Imaging conditions were identical to those used for AFD-expressed reporters. ASI and ASJ neurons were identified by soma position.

### Single molecule fluorescent in situ hybridization (smFISH)

The animals used were *pyt-1(oy169[pyt-1::gfp]); Ex[ttx-1p::tagRfp]*. Growth synchronized L1 larvae were cultivated at 20°C until the L3 stage. Worms were then shifted to different temperatures as indicated and fixed the next day as L4s. L4s were used because the thicker cuticle of 1 day old adults decreased staining efficacy. Stellaris gfp RNA FISH probes conjugated to Quasar 670 (Biosearch Technologies) were used. The Stellaris smFISH protocol was followed with the following modifications. After fixation, worms were permeabilized in 70% ethanol for 2 nights at 4°C. Hybridization was performed with probe concentration of 250 nM at 37°C overnight. No nuclear counterstain was used. Before mounting onto slides, samples were washed once with 2x SSC, then once with GLOX buffer, then suspended in GLOX buffer containing glucose oxidase and catalase. The sample was immediately used for imaging. Images were acquired on a Zeiss Axio Imager M2 epifluorescent microscope with a 63x oil objective (NA 1.4). z-stacks were acquired at 0.5 μm thickness. z-stacks were analyzed in ImageJ. AFD was identified using the AFD-specific *ttx-1p::tagRfp transgene*. No Quasar 670 signal was observed outside of AFD. Background subtraction was performed on the Quasar 670 channel, and puncta were quantified by scanning through the z-slices.

### Dauer formation assays

Dauer formation assays were performed as described previously (Neal et al., 2013; O’Donnell et al., 2018), with the following modifications. Assay plates were seeded with 10 μl of 16 mg/ml OP50. Live or heat-killed OP50 was used for 27°C-induced or pheromone-induced assays, respectively. Growth-synchronized adult hermaphrodites were allowed to lay 50–75 eggs per assay plate and plates were examined for dauers 70 hrs later. Pheromone-induced dauer formation assays were performed at 25°C using the ascr5 pheromone (Kaplan et al., 2011) at a final concentration of 600 nM in the plate agar. Dauer larvae were identified by their characteristic morphology.

### Motif enrichment analysis and identification of putative CREs

Transcription factor binding motifs represented as position weight matrices (PWMs) were acquired from a collection of CIS-BP database PWMs curated by MEME (Weirauch et al., 2014). The PWM set used in this analysis contained all available PWMs obtained for human, mouse, worm, and fly transcription factors (total of 2173 PWMs). This PWM set was then used as input for the SEA enrichment algorithm (Bailey and Grant, 2021) provided by the MEME suite of sequence analysis tools (Bailey et al., 2015), together with 500 bp DNA sequences immediately upstream of the start codons of each AFD-specific temperature-upregulated gene. Promoter sequences upstream of AFD-specific temperature-insensitive genes were used as control sequences. Enrichment by SEA was run with default parameters. SEA returned a list of 57 motifs that were over-represented in temperature-upregulated versus temperature-insensitive promoters. These motifs were then clustered by similarity into families of similar motifs using the matrix-clustering algorithm from RSAT (Castro-Mondragon, 2017) with the following parameters: –cor_th 0.7 –Ncor_th 0.5. To identify individual CREs, the promoters of a subset of temperature-upregulated genes were scanned using FIMO (Grant et al., 2011) for matches to any of the enriched CRE motifs identified by SEA. Highest scoring matches were chosen for perturbation via gene editing.

### Quantification and analyses

Summary plots of RNA-Seq data were generated with their associated packages in R, except for the plot of TRAP-Seq AFD expression versus CeNGEN AFD gene expression (Figure 1B), which was plotted in Prism 6 (GraphPad). Plots of fluorescence intensity, smFISH RNA puncta, dauer quantifications, T*AFD, and thermotaxis behavior quantifications were generated with Prism 6. GCaMP/FlincG3 fluorescence traces and thermotaxis assay example images were generated with MATLAB (Mathworks). Example images of fluorescent expression/localization reporters were made with ImageJ. The phylogenetic tree (Figure S5A) was generated with Geneious Prime (Biomatters). Multiple sequence alignment plots (Figure S3C, Figure S5B) were generated with Jalview 2.11.2.5 (Waterhouse et al., 2009). Statistical analyses were performed in Prism 6. Statistical test details, the number of analyzed samples and biologically independent experiments are reported in each figure legend.

## Supplementary Material

Supplement 1

## References

[1] Ailion M. and Thomas J. H.. Dauer formation induced by high temperatures in Caenorhabditis elegans. Genetics, 156(3):1047–1067, November 2000. Number: 3. 11063684 10.1093/genetics/156.3.1047 PMC1461313

[2] Aurelio Oscar, Hall David H., and Hobert Oliver. Immunoglobulin-domain proteins required for maintenance of ventral nerve cord organization. Science (New York, N.Y.), 295(5555):686–690, January 2002. Number: 5555. 11809975 10.1126/science.1066642

[3] Bailey Timothy L. and Grant Charles E.. SEA: Simple Enrichment Analysis of motifs, August 2021. Pages: 2021.08.23.457422 Section: New Results.

[4] Bailey Timothy L., Johnson James, Grant Charles E., and Noble William S.. The MEME Suite. Nucleic Acids Research, 43(Web Server issue):W39–W49, July 2015. Number: Web Server issue. 25953851 10.1093/nar/gkv416 PMC4489269

[5] Baldwin Maude W. and Ko Meng-Ching. Functional evolution of vertebrate sensory receptors. Hormones and Behavior, 124:104771, August 2020. 32437717 10.1016/j.yhbeh.2020.104771

[6] Bargmann Cornelia I.. Chemosensation in C. elegans. WormBook, October 2006. Publication Title: WormBook: The Online Review of C. elegans Biology [Internet]. 10.1895/wormbook.1.123.1 PMC4781564 18050433

[7] Beagan Jonathan A., Pastuzyn Elissa D., Fernandez Lindsey R., Guo Michael H., Feng Kelly, Titus Katelyn R., Chandrashekar Harshini, Shepherd Jason D., and Phillips-Cremins Jennifer E.. Three-dimensional genome restructuring across timescales of activity-induced neuronal gene expression. Nature Neuroscience, 23(6):707–717, June 2020. Number: 6. 32451484 10.1038/s41593-020-0634-6 PMC7558717

[8] Bear Daniel M., Lassance Jean-Marc, Hoekstra Hopi E., and Datta Sandeep Robert. The Evolving Neural and Genetic Architecture of Vertebrate Olfaction. Current biology: CB, 26(20):R1039–R1049, October 2016. Number: 20. 27780046 10.1016/j.cub.2016.09.011 PMC5104188

[9] Bhargava Yogesh, Hampden-Smith Kathryn, Konstantina Chachlaki, Wood Katherine C., Vernon Jeffrey, Allerston Charles K., Batchelor Andrew M., and Garthwaite John. Improved genetically-encoded, FlincG-type fluorescent biosensors for neural cGMP imaging. Frontiers in Molecular Neuroscience, 6:26, 2013. 24068983 10.3389/fnmol.2013.00026 PMC3781335

[10] Bliim Nicola, Leshchyns’ka Iryna, Sytnyk Vladimir, and Janitz Michael. Transcriptional regulation of long-term potentiation. Neurogenetics, 17(4):201–210, October 2016. Number: 4. 27318935 10.1007/s10048-016-0489-x

[11] Bourtchuladze R., Frenguelli B., Blendy J., Cioffi D., Schutz G., and Silva A. J.. Deficient long-term memory in mice with a targeted mutation of the cAMP-responsive element-binding protein. Cell, 79(1):59–68, October 1994. Number: 1. 7923378 10.1016/0092-8674(94)90400-6

[12] Burz D. S., Rivera-Pomar R., Jäckle H., and Hanes S. D.. Cooperative DNA-binding by Bicoid provides a mechanism for threshold-dependent gene activation in the Drosophila embryo. The EMBO journal, 17(20):5998–6009, October 1998. Number: 20. 9774343 10.1093/emboj/17.20.5998 PMC1170926

[13] Cantaut-Belarif Yasmine, Del’Immagine Adeline Orts, Penru Margot, Pézeron Guillaume, Wyart Claire, and Bardet Pierre-Luc. Adrenergic activation modulates the signal from the Reissner fiber to cerebrospinal fluid-contacting neurons during development. eLife, 9:e59469, October 2020. 33048048 10.7554/eLife.59469 PMC7591253

[14] Castro-Mondragon Jaime Abraham, Jaeger Sébastien, Thieffry Denis, Thomas-Chollier Morgane, and van Helden Jacques. RSAT matrix-clustering: dynamic exploration and redundancy reduction of transcription factor binding motif collections. Nucleic Acids Research, 45(13):e119, July 2017. Number: 13. 28591841 10.1093/nar/gkx314 PMC5737723

[15] Cembrowski Mark S. and Spruston Nelson. Heterogeneity within classical cell types is the rule: lessons from hippocampal pyramidal neurons. Nature Reviews. Neuroscience, 20(4):193–204, April 2019. Number: 4. 30778192 10.1038/s41583-019-0125-5

[16] Chen Yen-Chih, Chen Hung-Jhen, Tseng Wei-Chin, Hsu Jiun-Min, Huang Tzu-Ting, Chen Chun-Hao, and Pan Chun-Liang. A C. elegans Thermosensory Circuit Regulates Longevity through crh-1/CREB-Dependent flp-6 Neuropeptide Signaling. Developmental Cell, 39(2):209–223, October 2016. Number: 2. 27720609 10.1016/j.devcel.2016.08.021

[17] Clark Damon A., Biron David, Sengupta Piali, and Samuel Aravinthan D. T.. The AFD sensory neurons encode multiple functions underlying thermotactic behavior in Caenorhabditis elegans. The Journal of Neuroscience: The Official Journal of the Society for Neuroscience, 26(28):7444–7451, July 2006. Number: 28. 16837592 10.1523/JNEUROSCI.1137-06.2006 PMC6674189

[18] Colosimo Marc E., Brown Adam, Mukhopadhyay Saikat, Gabel Christopher, Lanjuin Anne E., Samuel Aravinthan D. T., and Sengupta Piali. Identification of thermosensory and olfactory neuron-specific genes via expression profiling of single neuron types. Current biology: CB, 14(24):2245–2251, December 2004. Number: 24. 15620651 10.1016/j.cub.2004.12.030

[19] Dobin Alexander, Davis Carrie A., Schlesinger Felix, Drenkow Jorg, Zaleski Chris, Jha Sonali, Batut Philippe, Chaisson Mark, and Gingeras Thomas R.. STAR: ultrafast universal RNA-seq aligner. Bioinformatics (Oxford, England), 29(1):15–21, January 2013. Number: 1. 23104886 10.1093/bioinformatics/bts635 PMC3530905

[20] Ernfors P., Lee K. F., and Jaenisch R.. Mice lacking brain-derived neurotrophic factor develop with sensory deficits. Nature, 368(6467):147–150, March 1994. Number: 6467. 8139657 10.1038/368147a0

[21] Douglas Fields R., Eshete Feleke, Stevens Beth, and Itoh Kouichi. Action Potential-Dependent Regulation of Gene Expression: Temporal Specificity in Ca2+, cAMP-Responsive Element Binding Proteins, and Mitogen-Activated Protein Kinase Signaling. Journal of Neuroscience, 17(19):7252–7266, October 1997. Publisher: Society for Neuroscience Section: Articles. 9295372 10.1523/JNEUROSCI.17-19-07252.1997 PMC6573446

[22] Fielenbach Nicole and Antebi Adam. C. elegans dauer formation and the molecular basis of plasticity. Genes & Development, 22(16):2149–2165, August 2008. Number: 16. 18708575 10.1101/gad.1701508 PMC2735354

[23] Flavell Steven W. and Greenberg Michael E.. Signaling mechanisms linking neuronal activity to gene expression and plasticity of the nervous system. Annual Review of Neuroscience, 31:563–590, 2008. 10.1146/annurev.neuro.31.060407.125631 PMC2728073 18558867

[24] Fowler Trent, Sen Ranjan, and Roy Ananda L.. Regulation of primary response genes. Molecular Cell, 44(3):348–360, November 2011. Number: 3. 22055182 10.1016/j.molcel.2011.09.014 PMC3212756

[25] Gershow Marc, Berck Matthew, Mathew Dennis, Luo Linjiao, Kane Elizabeth A., Carlson John R., and Samuel Aravinthan D. T.. Controlling airborne cues to study small animal navigation. Nature Methods, 9(3):290–296, January 2012. Number: 3. 22245808 10.1038/nmeth.1853 PMC3513333

[26] Ghanta Krishna S., Chen Zexiang, Mir Aamir, Dokshin Gregoriy A., Krishnamurthy Pranathi M., Yoon Yeonsoo, Gallant Judith, Xu Ping, Zhang Xiao-Ou, Ozturk Ahmet Rasit, Shin Masahiro, Idrizi Feston, Liu Pengpeng, Gneid Hassan, Edraki Alireza, Lawson Nathan D., Rivera-Pérez Jaime A., Sontheimer Erik J., Watts Jonathan K., and Mello Craig C.. 5’-Modifications improve potency and efficacy of DNA donors for precision genome editing. eLife, 10:e72216, October 2021. 34665130 10.7554/eLife.72216 PMC8568340

[27] Giorgetti Luca, Siggers Trevor, Tiana Guido, Caprara Greta, Notarbartolo Samuele, Corona Teresa, Pasparakis Manolis, Milani Paolo, Bulyk Martha L., and Natoli Gioacchino. Noncooperative interactions between transcription factors and clustered DNA binding sites enable graded transcriptional responses to environmental inputs. Molecular Cell, 37(3):418–428, February 2010. Number: 3. 20159560 10.1016/j.molcel.2010.01.016

[28] Golden J. W. and Riddle D. L.. The Caenorhabditis elegans dauer larva: developmental effects of pheromone, food, and temperature. Developmental Biology, 102(2):368–378, April 1984. Number: 2. 6706004 10.1016/0012-1606(84)90201-x

[29] Golden J. W. and Riddle D. L.. A pheromone-induced developmental switch in Caenorhabditis elegans: Temperature-sensitive mutants reveal a wild-type temperature-dependent process. Proceedings of the National Academy of Sciences of the United States of America, 81(3):819–823, February 1984. Number: 3. 6583682 10.1073/pnas.81.3.819 PMC344929

[30] Goodman Miriam B. and Sengupta Piali. How Caenorhabditis elegans Senses Mechanical Stress, Temperature, and Other Physical Stimuli. Genetics, 212(1):25–51, May 2019. Number: 1. 31053616 10.1534/genetics.118.300241 PMC6499529

[31] Gorla Madhavi, Chaudhari Karina, Hale Maya, Potter Chloe, and Bashaw Greg J.. A Nedd4 E3 Ubiquitin ligase pathway inhibits Robo1 repulsion and promotes commissural axon guidance across the midline. The Journal of Neuroscience: The Official Journal of the Society for Neuroscience, 42(40):7547–7561, August 2022. Number: 40. 36002265 10.1523/JNEUROSCI.2491-21.2022 PMC9546450

[32] Gorla Madhavi, Chaudhari Karina, Hale Maya, Potter Chloe, and Bashaw Greg J.. A Nedd4 E3 Ubiquitin Ligase Pathway Inhibits Robo1 Repulsion and Promotes Commissural Axon Guidance across the Midline. Journal of Neuroscience, 42(40):7547–7561, October 2022. Publisher: Society for Neuroscience Section: Research Articles. 36002265 10.1523/JNEUROSCI.2491-21.2022 PMC9546450

[33] Gracida Xicotencatl and Calarco John A.. Cell type-specific transcriptome profiling in C. elegans using the Translating Ribosome Affinity Purification technique. Methods (San Diego, Calif.), 126:130–137, August 2017. 28648677 10.1016/j.ymeth.2017.06.023

[34] Grant Charles E., Bailey Timothy L., and Noble William Stafford. FIMO: scanning for occurrences of a given motif. Bioinformatics, 27(7):1017–1018, April 2011. Number: 7. 21330290 10.1093/bioinformatics/btr064 PMC3065696

[35] Gray Jesse M. and Spiegel Ivo. Cell-type-specific programs for activity-regulated gene expression. Current Opinion in Neurobiology, 56:33–39, June 2019. 30529822 10.1016/j.conb.2018.11.001

[36] Gruner Matthew, Nelson Dru, Winbush Ari, Hintz Rebecca, Ryu Leesun, Chung Samuel H., Kim Kyuhyung, Gabel Chrisopher V., and van der Linden Alexander M.. Feeding state, insulin and NPR-1 modulate chemoreceptor gene expression via integration of sensory and circuit inputs. PLoS genetics, 10(10):e1004707, October 2014. Number: 10. 25357003 10.1371/journal.pgen.1004707 PMC4214617

[37] Harty R. N., Brown M. E., Wang G., Huibregtse J., and Hayes F. P.. A PPxY motif within the VP40 protein of Ebola virus interacts physically and functionally with a ubiquitin ligase: implications for filovirus budding. Proceedings of the National Academy of Sciences of the United States of America, 97(25):13871–13876, December 2000. Number: 25. 11095724 10.1073/pnas.250277297 PMC17668

[38] Hawk Josh D., Calvo Ana C., Liu Ping, Almoril-Porras Agustin, Aljobeh Ahmad, Torruella-Suárez María Luisa, Ren Ivy, Cook Nathan, Greenwood Joel, Luo Linjiao, Wang Zhao-Wen, Samuel Aravinthan D. T., and Daniel A. Colón-Ramos. Integration of Plasticity Mechanisms within a Single Sensory Neuron of C. elegans Actuates a Memory. Neuron, 97(2):356–367.e4, January 2018. Number: 2. 29307713 10.1016/j.neuron.2017.12.027 PMC5806692

[39] Hedgecock E. M. and Russell R. L.. Normal and mutant thermotaxis in the nematode Caenorhabditis elegans. Proceedings of the National Academy of Sciences of the United States of America, 72(10):4061–4065, October 1975. Number: 10. 1060088 10.1073/pnas.72.10.4061 PMC433138

[40] Heiman Myriam, Schaefer Anne, Gong Shiaoching, Peterson Jayms D., Day Michelle, Ramsey Keri E., Suárez-Fariñas Mayte, Schwarz Cordelia, Stephan Dietrich A., Surmeier D. James, Greengard Paul, and Heintz Nathaniel. A translational profiling approach for the molecular characterization of CNS cell types. Cell, 135(4):738–748, November 2008. Number: 4. 19013281 10.1016/j.cell.2008.10.028 PMC2696821

[41] Hettema Ewald H., Valdez-Taubas Javier, and Pelham Hugh R. B.. Bsd2 binds the ubiquitin ligase Rsp5 and mediates the ubiquitination of transmembrane proteins. The EMBO journal, 23(6):1279–1288, March 2004. Number: 6. 14988731 10.1038/sj.emboj.7600137 PMC381411

[42] Hobert Oliver. The neuronal genome of Caenorhabditis elegans. WormBook: The Online Review of C. Elegans Biology, pages 1–106, August 2013. 10.1895/wormbook.1.161.1 PMC4781646 24081909

[43] Hobert Oliver and Kratsios Paschalis. Neuronal identity control by terminal selectors in worms, flies, and chordates. Current Opinion in Neurobiology, 56:97–105, June 2019. 30665084 10.1016/j.conb.2018.12.006

[44] Hong Elizabeth J., McCord Alejandra E., and Greenberg Michael E.. A biological function for the neuronal activity-dependent component of Bdnf transcription in the development of cortical inhibition. Neuron, 60(4):610–624, November 2008. Number: 4. 19038219 10.1016/j.neuron.2008.09.024 PMC2873221

[45] Houchmandzadeh Bahram, Wieschaus Eric, and Leibler Stanislas. Establishment of developmental precision and proportions in the early Drosophila embryo. Nature, 415(6873):798–802, February 2002. Number: 6873. 11845210 10.1038/415798a

[46] Hrvatin Sinisa, Hochbaum Daniel R., Nagy M. Aurel, Cicconet Marcelo, Robertson Keiramarie, Cheadle Lucas, Zilionis Rapolas, Ratner Alex, Borges-Monroy Rebeca, Klein Allon M., Sabatini Bernardo L., and Greenberg Michael E.. Single-cell analysis of experience-dependent transcriptomic states in the mouse visual cortex. Nature Neuroscience, 21(1):120–129, January 2018. Number: 1. 29230054 10.1038/s41593-017-0029-5 PMC5742025

[47] Hu Peng, Fabyanic Emily, Kwon Deborah Y., Tang Sheng, Zhou Zhaolan, and Wu Hao. Dissecting Cell-Type Composition and Activity-Dependent Transcriptional State in Mammalian Brains by Massively Parallel Single-Nucleus RNA-Seq. Molecular Cell, 68(5):1006–1015.e7, December 2017. Number: 5. 29220646 10.1016/j.molcel.2017.11.017 PMC5743496

[48] Huang Shenq-Shyang, Hsu Li-Jin, and Chang Nan-Shan. Functional role of WW domain-containing proteins in tumor biology and diseases: Insight into the role in ubiquitin-proteasome system. FASEB bioAdvances, 2(4):234–253, April 2020. Number: 4. 32259050 10.1096/fba.2019-00060 PMC7133736

[49] Huber Armin. Scaffolding proteins organize multimolecular protein complexes for sensory signal transduction. European Journal of Neuroscience, 14(5):769–776, 2001. Number: 5 _eprint: 10.1046/j.0953-816x.2001.01704.x. 11576180

[50] Hölter Philip, Kunst Stefanie, Wolloscheck Tanja, Kelleher Debra K., Sticht Carsten, Wolfrum Uwe, and Spessert Rainer. The retinal clock drives the expression of Kcnv2, a channel essential for visual function and cone survival. Investigative Ophthalmology & Visual Science, 53(11):6947–6954, October 2012. Number: 11. 22969075 10.1167/iovs.12-10234

[51] Inada Hitoshi, Ito Hiroko, Satterlee John, Sengupta Piali, Matsumoto Kunihiro, and Mori Ikue. Identification of guanylyl cyclases that function in thermosensory neurons of Caenorhabditis elegans. Genetics, 172(4):2239–2252, April 2006. Number: 4. 16415369 10.1534/genetics.105.050013 PMC1456394

[52] Ippolito Domenica, Thapliyal Saurabh, and Glauser Dominique A. Ca2+/CaM binding to CaMKI promotes IMA-3 importin binding and nuclear translocation in sensory neurons to control behavioral adaptation. eLife, 10:e71443, November 2021. Publisher: eLife Sciences Publications, Ltd. 34766550 10.7554/eLife.71443 PMC8635976

[53] Ji Ni, Venkatachalam Vivek, Denise Rodgers Hillary, Hung Wesley, Kawano Taizo, Clark Christopher M., Lim Maria, Alkema Mark J., Zhen Mei, and Samuel Aravinthan Dt. Corollary discharge promotes a sustained motor state in a neural circuit for navigation. eLife, 10:e68848, April 2021. 33880993 10.7554/eLife.68848 PMC8139836

[54] Jones K. R., Fariñas I., Backus C., and Reichardt L. F.. Targeted disruption of the BDNF gene perturbs brain and sensory neuron development but not motor neuron development. Cell, 76(6):989–999, March 1994. Number: 6. 8137432 10.1016/0092-8674(94)90377-8 PMC2711896

[55] Julius David and Nathans Jeremy. Signaling by sensory receptors. Cold Spring Harbor Perspectives in Biology, 4(1):a005991, January 2012. Number: 1. 22110046 10.1101/cshperspect.a005991 PMC3249628

[56] Kaang B. K., Kandel E. R., and Grant S. G.. Activation of cAMP-responsive genes by stimuli that produce long-term facilitation in Aplysia sensory neurons. Neuron, 10(3):427–435, March 1993. Number: 3. 8384857 10.1016/0896-6273(93)90331-k

[57] Kaletsky Rachel, Lakhina Vanisha, Arey Rachel, Williams April, Landis Jessica, Ashraf Jasmine, and Murphy Coleen T.. The C. elegans adult neuronal IIS/FOXO transcriptome reveals adult phenotype regulators. Nature, 529(7584):92–96, January 2016. Number: 7584. 26675724 10.1038/nature16483 PMC4708089

[58] Kaplan Fatma, Srinivasan Jagan, Mahanti Parag, Ajredini Ramadan, Durak Omer, Nimalendran Rathika, Sternberg Paul W., Teal Peter E. A., Schroeder Frank C., Edison Arthur S., and Alborn Hans T.. Ascaroside expression in Caenorhabditis elegans is strongly dependent on diet and developmental stage. PloS One, 6(3):e17804, March 2011. Number: 3. 21423575 10.1371/journal.pone.0017804 PMC3058051

[59] Kaye Julia A., Rose Natalie C., Goldsworthy Brett, Goga Andrei, and L’Etoile Noelle D.. A 3’UTR pumilio-binding element directs translational activation in olfactory sensory neurons. Neuron, 61(1):57–70, January 2009. Number: 1. 19146813 10.1016/j.neuron.2008.11.012 PMC4274156

[60] Kimura Koutarou D., Miyawaki Atsushi, Matsumoto Kunihiro, and Mori Ikue. The C. elegans thermosensory neuron AFD responds to warming. Current biology: CB, 14(14):1291–1295, July 2004. Number: 14. 15268861 10.1016/j.cub.2004.06.060

[61] Klass Michael R.. Aging in the nematode Caenorhabditis elegans: Major biological and environmental factors influencing life span. Mechanisms of Ageing and Development, 6:413–429, January 1977. 926867 10.1016/0047-6374(77)90043-4

[62] Kobayashi Kyogo, Nakano Shunji, Amano Mutsuki, Tsuboi Daisuke, Nishioka Tomoki, Ikeda Shingo, Yokoyama Genta, Kaibuchi Kozo, and Mori Ikue. Single-Cell Memory Regulates a Neural Circuit for Sensory Behavior. Cell Reports, 14(1):11–21, January 2016. Number: 1. 26725111 10.1016/j.celrep.2015.11.064

[63] Korenbrot J. I. and Fernald R. D.. Circadian rhythm and light regulate opsin mRNA in rod photoreceptors. Nature, 337(6206):454–457, February 1989. Number: 6206. 2521689 10.1038/337454a0

[64] Kulik Yelena, Jones Ryan, Moughamian Armen J., Whippen Jenna, and Davis Graeme W.. Dual separable feedback systems govern firing rate homeostasis. eLife, 8:e45717, April 2019. 30973325 10.7554/eLife.45717 PMC6491091

[65] Lacar Benjamin, Linker Sara B., Jaeger Baptiste N., Krishnaswami Suguna R., Barron Jerika J., Kelder Martijn J. E., Parylak Sarah L., Paquola Apuã C. M., Venepally Pratap, Novotny Mark, O’Connor Carolyn, Fitzpatrick Conor, Erwin Jennifer A., Hsu Jonathan Y., Husband David, McConnell Michael J., Lasken Roger, and Gage Fred H.. Nuclear RNA-seq of single neurons reveals molecular signatures of activation. Nature Communications, 7:11022, April 2016. 10.1038/ncomms11022 PMC4838832 27090946

[66] Lee Yong-Seok, Bailey Craig H., Kandel Eric R., and Kaang Bong-Kiun. Transcriptional regulation of long-term memory in the marine snail Aplysia. Molecular Brain, 1:3, June 2008. 18803855 10.1186/1756-6606-1-3 PMC2546398

[67] Love Michael I., Huber Wolfgang, and Anders Simon. Moderated estimation of fold change and dispersion for RNA-seq data with DESeq2. Genome Biology, 15(12):550, December 2014. Number: 12. 25516281 10.1186/s13059-014-0550-8 PMC4302049

[68] Luo Linjiao, Cook Nathan, Venkatachalam Vivek, Martinez-Velazquez Luis A., Zhang Xiaodong, Calvo Ana C., Hawk Josh, MacInnis Bronwyn L., Frank Michelle, Ng Jia Hong Ray, Klein Mason, Gershow Marc, Hammarlund Marc, Goodman Miriam B., Colón-Ramos Daniel A., Zhang Yun, and Samuel Aravinthan D. T.. Bidirectional thermotaxis in Caenorhabditis elegans is mediated by distinct sensorimotor strategies driven by the AFD thermosensory neurons. Proceedings of the National Academy of Sciences of the United States of America, 111(7):2776–2781, February 2014. Number: 7. 24550307 10.1073/pnas.1315205111 PMC3932917

[69] Mardinly A. R., Spiegel I., Patrizi A., Centofante E., Bazinet J. E., Tzeng C. P., Mandel-Brehm C., Harmin D. A., Adesnik H., Fagiolini M., and Greenberg M. E.. Sensory experience regulates cortical inhibition by inducing IGF1 in VIP neurons. Nature, 531(7594):371–375, March 2016. Number: 7594. 26958833 10.1038/nature17187 PMC4823817

[70] Martelli Carlotta and Storace Douglas Anthony. Stimulus Driven Functional Transformations in the Early Olfactory System. Frontiers in Cellular Neuroscience, 15:684742, 2021. 34413724 10.3389/fncel.2021.684742 PMC8369031

[71] Martin Marcel. Cutadapt removes adapter sequences from high-throughput sequencing reads. EMB-net.journal, 17(1):10–12, May 2011. Number: 1.

[72] McMahon Douglas G., Iuvone P. Michael, and Tosini Gianluca. Circadian organization of the mammalian retina: from gene regulation to physiology and diseases. Progress in Retinal and Eye Research, 39:58–76, March 2014. 24333669 10.1016/j.preteyeres.2013.12.001 PMC3927986

[73] Meisel Joshua D., Panda Oishika, Mahanti Parag, Schroeder Frank C., and Kim Dennis H.. Chemosensation of bacterial secondary metabolites modulates neuroendocrine signaling and behavior of C. elegans. Cell, 159(2):267–280, October 2014. Number: 2. 25303524 10.1016/j.cell.2014.09.011 PMC4194030

[74] Myat Anna, Henry Pauline, McCabe Veronica, Flintoft Louisa, Rotin Daniela, and Tear Guy. Drosophila Nedd4, a ubiquitin ligase, is recruited by Commissureless to control cell surface levels of the roundabout receptor. Neuron, 35(3):447–459, August 2002. Number: 3. 12165468 10.1016/s0896-6273(02)00795-x

[75] Myat Anna, Henry Pauline, McCabe Veronica, Flintoft Louisa, Rotin Daniela, and Tear Guy. Drosophila Nedd4, a Ubiquitin Ligase, Is Recruited by Commissureless to Control Cell Surface Levels of the Roundabout Receptor. Neuron, 35(3):447–459, August 2002. 12165468 10.1016/s0896-6273(02)00795-x

[76] Nakano Shunji, Nakayama Airi, Kuroyanagi Hiroo, Yamashiro Riku, Tsukada Yuki, and Mori Ikue. Genetic screens identified dual roles of MAST kinase and CREB within a single thermosensory neuron in the regulation of C. elegans thermotaxis behavior. G3 (Bethesda, Md.), page jkac248, September 2022. 36102820 10.1093/g3journal/jkac248 PMC9635662

[77] Neal Scott J., Kim Kyuhyung, and Sengupta Piali. Quantitative assessment of pheromone-induced Dauer formation in Caenorhabditis elegans. Methods in Molecular Biology (Clifton, N.J.), 1068:273–283, 2013. 10.1007/978-1-62703-619-1_20 24014369

[78] Nechipurenko Inna V., Olivier-Mason Anique, Kazatskaya Anna, Kennedy Julie, McLachlan Ian G., Heiman Maxwell G., Blacque Oliver E., and Sengupta Piali. A Conserved Role for Girdin in Basal Body Positioning and Ciliogenesis. Developmental Cell, 38(5):493–506, September 2016. Number: 5. 27623382 10.1016/j.devcel.2016.07.013 PMC5023068

[79] Nguyen Phuong Anh T., Liou Willisa, Hall David H., and Leroux Michel R.. Ciliopathy proteins establish a bipartite signaling compartment in a C. elegans thermosensory neuron. Journal of Cell Science, 127(Pt 24):5317–5330, December 2014. Number: Pt 24. 25335890 10.1242/jcs.157610 PMC4265742

[80] Nishida Yukuo, Sugi Takuma, Nonomura Mayu, and Mori Ikue. Identification of the AFD neuron as the site of action of the CREB protein in Caenorhabditis elegans thermotaxis. EMBO reports, 12(8):855–862, July 2011. Number: 8. 21738224 10.1038/embor.2011.120 PMC3147260

[81] O’Donnell Michael P., Chao Pin-Hao, Kammenga Jan E., and Sengupta Piali. Rictor/TORC2 mediates gut-to-brain signaling in the regulation of phenotypic plasticity in C. elegans. PLoS genetics, 14(2):e1007213, February 2018. Number: 2. 29415022 10.1371/journal.pgen.1007213 PMC5819832

[82] Ohnishi Noriyuki, Kuhara Atsushi, Nakamura Fumiya, Okochi Yoshifumi, and Mori Ikue. Bidirectional regulation of thermotaxis by glutamate transmissions in Caenorhabditis elegans. The EMBO journal, 30(7):1376–1388, April 2011. Number: 7. 21304490 10.1038/emboj.2011.13 PMC3094115

[83] O’Leary Timothy, Williams Alex H., Franci Alessio, and Marder Eve. Cell types, network homeostasis, and pathological compensation from a biologically plausible ion channel expression model. Neuron, 82(4):809–821, May 2014. Number: 4. 24853940 10.1016/j.neuron.2014.04.002 PMC4109293

[84] Park JiSoo, Oh Hyekyoung, Kim Do-Young, Cheon YongJin, Park Yeon-Ji, Hwang Hyeonjeong, Neal Scott J., Dar Abdul Rouf, Butcher Rebecca A., Sengupta Piali, Kim Dae-Won, and Kim Kyuhyung. CREB mediates the C. elegans dauer polyphenism through direct and cell-autonomous regulation of TGF-β expression. PLoS genetics, 17(7):e1009678, July 2021. Number: 7. 34260587 10.1371/journal.pgen.1009678 PMC8312985

[85] Peckol E. L., Troemel E. R., and Bargmann C. I.. Sensory experience and sensory activity regulate chemosensory receptor gene expression in Caenorhabditis elegans. Proceedings of the National Academy of Sciences of the United States of America, 98(20):11032–11038, September 2001. Number: 20. 11572964 10.1073/pnas.191352498 PMC58678

[86] Peng Hanchuan, Xie Peng, Liu Lijuan, Kuang Xiuli, Wang Yimin, Qu Lei, Gong Hui, Jiang Shengdian, Li Anan, Ruan Zongcai, Ding Liya, Yao Zizhen, Chen Chao, Chen Mengya, Daigle Tanya L., Dalley Rachel, Ding Zhangcan, Duan Yanjun, Feiner Aaron, He Ping, Hill Chris, Hirokawa Karla E., Hong Guodong, Huang Lei, Kebede Sara, Kuo Hsien-Chi, Larsen Rachael, Lesnar Phil, Li Longfei, Li Qi, Li Xiangning, Li Yaoyao, Li Yuanyuan, Liu An, Lu Donghuan, Mok Stephanie, Ng Lydia, Thuc Nghi Nguyen Qiang Ouyang, Pan Jintao, Shen Elise, Song Yuanyuan, Sunkin Susan M., Tasic Bosiljka, Veldman Matthew B., Wakeman Wayne, Wan Wan, Wang Peng, Wang Quanxin, Wang Tao, Wang Yaping, Xiong Feng, Xiong Wei, Xu Wenjie, Ye Min, Yin Lulu, Yu Yang, Yuan Jia, Yuan Jing, Yun Zhixi, Zeng Shaoqun, Zhang Shichen, Zhao Sujun, Zhao Zijun, Zhou Zhi, Huang Z. Josh, Esposito Luke, Hawrylycz Michael J., Sorensen Staci A., Yang X. William, Zheng Yefeng, Gu Zhongze, Xie Wei, Koch Christof, Luo Qingming, Harris Julie A., Wang Yun, and Zeng Hongkui. Morphological diversity of single neurons in molecularly defined cell types. Nature, 598(7879):174–181, October 2021. Number: 7879. 34616072 10.1038/s41586-021-03941-1 PMC8494643

[87] Pugh E. N., Nikonov S., and Lamb T. D.. Molecular mechanisms of vertebrate photoreceptor light adaptation. Current Opinion in Neurobiology, 9(4):410–418, August 1999. Number: 4. 10448166 10.1016/S0959-4388(99)80062-2

[88] Ramot Daniel, MacInnis Bronwyn L., and Goodman Miriam B.. Bidirectional temperature-sensing by a single thermosensory neuron in C. elegans. Nature Neuroscience, 11(8):908–915, August 2008. Number: 8. 18660808 10.1038/nn.2157 PMC2587641

[89] Roselli Camilla, Ramaswami Mani, Boto Tamara, and Cervantes-Sandoval Isaac. The Making of Long-Lasting Memories: A Fruit Fly Perspective. Frontiers in Behavioral Neuroscience, 15:662129, 2021. 33859556 10.3389/fnbeh.2021.662129 PMC8042140

[90] Rotin D., Kanelis V., and Schild L.. Trafficking and cell surface stability of ENaC. American Journal of Physiology. Renal Physiology, 281(3):F391–399, September 2001. Number: 3. 11502587 10.1152/ajprenal.2001.281.3.F391

[91] Ryan Deborah A., Miller Renee M., Lee KyungHwa, Neal Scott J., Fagan Kelli A., Sengupta Piali, and Portman Douglas S.. Sex, age, and hunger regulate behavioral prioritization through dynamic modulation of chemoreceptor expression. Current biology: CB, 24(21):2509–2517, November 2014. Number: 21. 25438941 10.1016/j.cub.2014.09.032 PMC4254623

[92] Salah Zaidoun, Alian Akram, and Aqeilan Rami I.. WW domain-containing proteins: retrospectives and the future. Frontiers in Bioscience (Landmark Edition), 17(1):331–348, January 2012. Number: 1. 22201747 10.2741/3930

[93] Satterlee John S., Ryu William S., and Sengupta Piali. The CMK-1 CaMKI and the TAX-4 Cyclic Nucleotide-Gated Channel Regulate Thermosensory Neuron Gene Expression and Function in C. elegans. Current Biology, 14(1):62–68, January 2004. Number: 1. 14711416 10.1016/j.cub.2003.12.030

[94] Satterlee John S., Sasakura Hiroyuki, Kuhara Atsushi, Berkeley Maura, Mori Ikue, and Sengupta Piali. Specification of Thermosensory Neuron Fate in C. elegans Requires ttx-1, a Homolog of otd/Otx. Neuron, 31(6):943–956, September 2001. Number: 6. 11580895 10.1016/s0896-6273(01)00431-7

[95] Scala Federico, Kobak Dmitry, Bernabucci Matteo, Bernaerts Yves, Cadwell Cathryn René, Castro Jesus Ramon, Hartmanis Leonard, Jiang Xiaolong, Laturnus Sophie, Miranda Elanine, Mulherkar Shalaka, Tan Zheng Huan, Yao Zizhen, Zeng Hongkui, Sandberg Rickard, Berens Philipp, and Tolias Andreas S.. Phenotypic variation of transcriptomic cell types in mouse motor cortex. Nature, 598(7879):144–150, October 2021. Number: 7879. 33184512 10.1038/s41586-020-2907-3 PMC8113357

[96] Servello Francesco A., Fernandes Rute, Eder Matthias, Harris Nathan, Martin Olivier M. F., Oswal Natasha, Lindberg Anders, Derosiers Nohelly, Sengupta Piali, Stroustrup Nicholas, and Apfeld Javier. Neuronal temperature perception induces specific defenses that enable *C. elegans* to cope with the enhanced reactivity of hydrogen peroxide at high temperature. eLife, 11:e78941, October 2022. 36226814 10.7554/eLife.78941 PMC9635881

[97] Sheng M., McFadden G., and Greenberg M. E.. Membrane depolarization and calcium induce c-fos transcription via phosphorylation of transcription factor CREB. Neuron, 4(4):571–582, April 1990. Number: 4. 2157471 10.1016/0896-6273(90)90115-v

[98] Shepherd Jason D., Rumbaugh Gavin, Wu Jing, Chowdhury Shoaib, Plath Niels, Kuhl Dietmar, Huganir Richard L., and Worley Paul F.. Arc/Arg3.1 mediates homeostatic synaptic scaling of AMPA receptors. Neuron, 52(3):475–484, November 2006. Number: 3. 17088213 10.1016/j.neuron.2006.08.034 PMC1764219

[99] Sims Jennie R., Ow Maria C., Nishiguchi Mailyn A., Kim Kyuhyung, Sengupta Piali, and Hall Sarah E.. Developmental programming modulates olfactory behavior in C. elegans via endogenous RNAi pathways. eLife, 5:e11642, June 2016. 27351255 10.7554/eLife.11642 PMC4924998

[100] Spiegel Ivo, Mardinly Alan R., Gabel Harrison W., Bazinet Jeremy E., Couch Cameron H., Tzeng Christopher P., Harmin David A., and Greenberg Michael E.. Npas4 regulates excitatory-inhibitory balance within neural circuits through cell-type-specific gene programs. Cell, 157(5):1216–1229, May 2014. Number: 5. 24855953 10.1016/j.cell.2014.03.058 PMC4089405

[101] Sternberg Jenna R., Prendergast Andrew E., Brosse Lucie, Cantaut-Belarif Yasmine, Thouvenin Olivier, Orts-Del’Immagine Adeline, Castillo Laura, Djenoune Lydia, Kurisu Shusaku, McDearmid Jonathan R., Bardet Pierre-Luc, Boccara Claude, Okamoto Hitoshi, Delmas Patrick, and Wyart Claire. Pkd2l1 is required for mechanoception in cerebrospinal fluid-contacting neurons and maintenance of spine curvature. Nature Communications, 9(1):3804, September 2018. Number: 1. 10.1038/s41467-018-06225-x PMC6143598 30228263

[102] Stewart-Ornstein Jacob, Nelson Christopher, De-Risi Joe, Weissman Jonathan S., and El-Samad Hana. Msn2 coordinates a stoichiometric gene expression program. Current biology: CB, 23(23):2336–2345, December 2013. Number: 23. 24210615 10.1016/j.cub.2013.09.043 PMC4072881

[103] Storch Kai-Florian, Paz Carlos, Signorovitch James, Raviola Elio, Pawlyk Basil, Li Tiansen, and Weitz Charles J.. Intrinsic circadian clock of the mammalian retina: importance for retinal processing of visual information. Cell, 130(4):730–741, August 2007. Number: 4. 17719549 10.1016/j.cell.2007.06.045 PMC2040024

[104] Takeishi Asuka, Yeon Jihye, Harris Nathan, Yang Wenxing, and Sengupta Piali. Feeding state functionally reconfigures a sensory circuit to drive thermosensory behavioral plasticity. eLife, 9:e61167, October 2020. 33074105 10.7554/eLife.61167 PMC7644224

[105] Takeishi Asuka, Yu Yanxun V., Hapiak Vera M., Bell Harold W., Timothy O’Leary, and Piali Sengupta. Receptor-type Guanylyl Cyclases Confer Thermosensory Responses in C. elegans. Neuron, 90(2):235–244, April 2016. Number: 2. 27041501 10.1016/j.neuron.2016.03.002 PMC4840083

[106] Taylor Seth R., Santpere Gabriel, Weinreb Alexis, Barrett Alec, Reilly Molly B., Xu Chuan, Varol Erdem, Oikonomou Panos, Glenwinkel Lori, McWhirter Rebecca, Poff Abigail, Basavaraju Manasa, Rafi Ib-nul, Yemini Eviatar, Cook Steven J., Abrams Alexander, Vidal Berta, Cros Cyril, Tavazoie Saeed, Sestan Nenad, Hammarlund Marc, Hobert Oliver, and Miller David M.. Molecular topography of an entire nervous system. Cell, 184(16):4329–4347.e23, August 2021. Number: 16. 34237253 10.1016/j.cell.2021.06.023 PMC8710130

[107] Thapliyal Saurabh, Beets Isabel, and Glauser Dominique A.. Multisite gating in tonic sensory circuits integrates multimodal context to control persistent behavioral states, July 2022. Pages: 2022.07.14.500040 Section: New Results. 10.1038/s41467-023-38685-1 PMC10220067 37236963

[108] Tsukahara Tatsuya, Brann David H., Pashkovski Stan L., Guitchounts Grigori, Bozza Thomas, and Sandeep Robert Datta. A transcriptional rheostat couples past activity to future sensory responses. Cell, 184(26):6326–6343.e32, December 2021. Number: 26. 34879231 10.1016/j.cell.2021.11.022 PMC8758202

[109] Tyssowski Kelsey M., DeStefino Nicholas R., Cho Jin-Hyung, Dunn Carissa J., Poston Robert G., Carty Crista E., Jones Richard D., Chang Sarah M., Romeo Palmyra, Wurzelmann Mary K., Ward James M., Andermann Mark L., Saha Ramendra N., Dudek Serena M., and Gray Jesse M.. Different Neuronal Activity Patterns Induce Different Gene Expression Programs. Neuron, 98(3):530–546.e11, May 2018. Number: 3. 29681534 10.1016/j.neuron.2018.04.001 PMC5934296

[110] Tyssowski Kelsey M. and Gray Jesse M.. The neuronal stimulation-transcription coupling map. Current Opinion in Neurobiology, 59:87–94, December 2019. 31163285 10.1016/j.conb.2019.05.001 PMC6885097

[111] Valperga Giulio and de Bono Mario. Impairing one sensory modality enhances another by reconfiguring peptidergic signalling in Caenorhabditis elegans. eLife, 11:e68040, February 2022. 35201977 10.7554/eLife.68040 PMC8871372

[112] Waterhouse Andrew M., Procter James B., Martin David M. A., Clamp Michèle, and Barton Geoffrey J.. Jalview Version 2–a multiple sequence alignment editor and analysis workbench. Bioinformatics (Oxford, England), 25(9):1189–1191, May 2009. Number: 9. 19151095 10.1093/bioinformatics/btp033 PMC2672624

[113] Weirauch Matthew T., Yang Ally, Albu Mihai, Cote Atina G., Montenegro-Montero Alejandro, Drewe Philipp, Najafabadi Hamed S., Lambert Samuel A., Mann Ishminder, Cook Kate, Zheng Hong, Goity Alejandra, Harm van Bakel Jean-Claude Lozano, Galli Mary, Lewsey Mathew G., Huang Eryong, Mukherjee Tuhin, Chen Xiaoting, ReeceHoyes John S., Govindarajan Sridhar, Shaulsky Gad, Walhout Albertha J. M., Bouget François-Yves, Ratsch Gunnar, Larrondo Luis F., Ecker Joseph R., and Hughes Timothy R.. Determination and inference of eukaryotic transcription factor sequence specificity. Cell, 158(6):1431–1443, September 2014. Number: 6. 25215497 10.1016/j.cell.2014.08.009 PMC4163041

[114] West A. E., Chen W. G., Dalva M. B., Dolmetsch R. E., Kornhauser J. M., Shaywitz A. J., Takasu M. A., Tao X., and Greenberg M. E.. Calcium regulation of neuronal gene expression. Proceedings of the National Academy of Sciences of the United States of America, 98(20):11024–11031, September 2001. Number: 20. 11572963 10.1073/pnas.191352298 PMC58677

[115] White J. G., Southgate E., Thomson J. N., and Brenner S.. The structure of the nervous system of the nematode Caenorhabditis elegans. Philosophical Transactions of the Royal Society of London. Series B, Biological Sciences, 314(1165):1–340, November 1986. Number: 1165. 22462104 10.1098/rstb.1986.0056

[116] Woldemariam Sarah, Nagpal Jatin, Hill Tyler, Li Joy, Schneider Martin W., Shankar Raakhee, Futey Mary, Varshney Aruna, Ali Nebat, Mitchell Jordan, Andersen Kristine, Barsi-Rhyne Benjamin, Tran Alan, Steuer Costa Wagner, Krzyzanowski Michelle C., Yu Yanxun V., Brueggemann Chantal, Hamilton O. Scott, Ferkey Denise M., VanHoven Miri, Sengupta Piali, Gottschalk Alexander, and L’Etoile Noelle. Using a Robust and Sensitive GFP-Based cGMP Sensor for Real-Time Imaging in Intact Caenorhabditis elegans. Genetics, 213(1):59–77, September 2019. Number: 1. 31331946 10.1534/genetics.119.302392 PMC6727795

[117] Wu Ye Emily, Pan Lin, Zuo Yanning, Li Xinmin, and Hong Weizhe. Detecting Activated Cell Populations Using Single-Cell RNA-Seq. Neuron, 96(2):313–329.e6, October 2017. Number: 2. 29024657 10.1016/j.neuron.2017.09.026

[118] Yap Ee-Lynn and Greenberg Michael E.. Activity-Regulated Transcription: Bridging the Gap between Neural Activity and Behavior. Neuron, 100(2):330–348, October 2018. Number: 2. 30359600 10.1016/j.neuron.2018.10.013 PMC6223657

[119] Yap Ee-Lynn, Pettit Noah L., Davis Christopher P., Nagy M. Aurel, Harmin David A., Golden Emily, Dagliyan Onur, Lin Cindy, Rudolph Stephanie, Sharma Nikhil, Griffith Eric C., Harvey Christopher D., and Greenberg Michael E.. Bidirectional perisomatic inhibitory plasticity of a Fos neuronal network. Nature, 590(7844):115–121, February 2021. Number: 7844. 33299180 10.1038/s41586-020-3031-0 PMC7864877

[120] Yau King-Wai and Hardie Roger C.. Phototransduction motifs and variations. Cell, 139(2):246–264, October 2009. Number: 2. 19837030 10.1016/j.cell.2009.09.029 PMC2885920

[121] Yu Guangchuang, Wang Li-Gen, Han Yanyan, and He Qing-Yu. clusterProfiler: an R Package for Comparing Biological Themes Among Gene Clusters. OMICS: A Journal of Integrative Biology, 16(5):284–287, May 2012. Number: 5 Publisher: Mary Ann Liebert, Inc., publishers. 22455463 10.1089/omi.2011.0118 PMC3339379

[122] Yu Yanxun V., Bell Harold W., Glauser Dominique, Van Hooser Stephen D., Goodman Miriam B., and Sengupta Piali. CaMKI-dependent regulation of sensory gene expression mediates experience-dependent plasticity in the operating range of a thermosensory neuron. Neuron, 84(5):919–926, December 2014. Number: 5. 25467978 10.1016/j.neuron.2014.10.046 PMC4258139

[123] Zang Jingjing and Neuhauss Stephan C. F.. The Binding Properties and Physiological Functions of Recoverin. Frontiers in Molecular Neuroscience, 11:473, 2018. 30618620 10.3389/fnmol.2018.00473 PMC6306944

[124] Zeng Hongkui. What is a cell type and how to define it? Cell, 185(15):2739–2755, July 2022. Number: 15. 35868277 10.1016/j.cell.2022.06.031 PMC9342916

