# Supplementary material for: Molecular encoding of stimulus features in a single sensory neuron type enables neuronal and behavioral plasticity
Source: bioRxiv. 2023 Jan 22:2023.01.22.525070. Preprint. [Version 1] doi: 10.1101/2023.01.22.525070 (PMC9882311; doi:10.1101/2023.01.22.525070)
Supplement: Supplement 1 [file NIHPP2023.01.22.525070v1-supplement-1.pdf]

bioRxiv preprint doi: https://doi.org/10.1101/2023.01.22.525070; this version posted January 22, 2023. The copyright holder for this preprint (which was not certified by peer review) is the author/funder, who has granted bioRxiv a license to display the preprint in perpetuity. It is made available under aCC-BY 4.0 International license.

Figure S1. Analysis of TRAP-Seq data (Related to Figure 1).

A) DESeq2-generated principal component analysis plot of TRAP-Seq data clustered by tissue and temperature cultivation condition. Each dot represents a single replicate.

B) Waterfall plot of temperature-dependent expression of genes in AFD encoding selected protein classes. Displayed genes exhibit > 2 or < -2 $\log_2$ fold change with a threshold of adjusted p-value < 0.05. Genes in the "Signal transduction" category were filtered by the gene ontology term "signal transduction" – GO:0007165. The categories "Transcription factors" and "Neuropeptides" were derived from a curated list of *C. elegans* transcription factors and neuronal genes (Hobert, 2013; Valperga and de Bono, 2022).

C) Enriched neuronal gene classes from temperature-dependent genes in AFD and whole animal samples using clusterProfiler (Yu et al., 2012) on curated *C. elegans* neuronal genes (Hobert, 2013; Valperga and de Bono, 2022).

Figure S2. Temperature-regulated expression of reporter fusion genes in AFD (Related to Figure 2).

A) (Left) Genomic structures of endogenously reporter tagged genes examined in Figure 2. (Right) Structures of promoter::GFP fusion reporters for an additional subset of genes. Expression from these genes were examined from extrachromosomal arrays in transgenic animals (Table S1). Light and dark gray boxes indicate untranslated regions and coding DNA, respectively.

B,C) (Left) Representative images of *F23D12.*3p::GFP (B) and *gcy-29*p::GFP (C) expression in AFD soma of adult animals grown at the indicated conditions. Scale bar: 5 µm. (Right) Quantification of GFP levels in adult animals grown at the indicated conditions. Each dot is a

bioRxiv preprint doi: https://doi.org/10.1101/2023.01.22.525070; this version posted January 22, 2023. The copyright holder for this preprint (which was not certified by peer review) is the author/funder, who has granted bioRxiv a license to display the preprint in perpetuity. It is made available under aCC-BY 4.0 International license.

measurement from a single AFD neuron. Horizontal and vertical lines indicate mean and SD, respectively. n = 33-55 neurons from at least two biologically independent experiments.

D) Quantification of DAC-1::GFP levels in a hypodermal cell (left) or unidentified head neuron in adult animals grown at the indicated conditions. Each dot is a measurement from a single cell. Horizontal and vertical lines indicate mean and SD, respectively. n = 12-19 cells from at least two biologically independent experiments.

For all panels, *** indicates different at p<0.001 (t test or one-way ANOVA with Dunnett's multiple comparisons correction); ns – not significant.

Figure S3. Analysis of *cis*-regulatory sequences upstream of *pyt-1* and *dac-1* (Related to Figure 5).

A) *dac-1* genomic locus showing the a and b isoforms. Grey and black boxes indicate untranslated regions and coding DNA, respectively. The structures of the transcriptional reporter genes examined are shown below.

B) (Left) Representative images of GFP driven by the *dac-1b* promoter in adult animals grown at the indicated conditions. Scale bar: 5 µm. (Right) Quantification of *dac-1b*p::GFP levels in adult animals grown at the indicated conditions. Each dot is a measurement from a single AFD neuron. Horizontal and vertical lines indicate mean and SD, respectively. n = 27-33 neurons from at least two biologically independent experiments. All conditions were assayed in parallel; data from 15°C overnight grown animals are repeated in the two panels. ** and *** indicate different at p<0.01 and 0.001, respectively (one-way ANOVA with Dunnett's multiple comparisons correction).

bioRxiv preprint doi: https://doi.org/10.1101/2023.01.22.525070; this version posted January 22, 2023. The copyright holder for this preprint (which was not certified by peer review) is the author/funder, who has granted bioRxiv a license to display the preprint in perpetuity. It is made available under aCC-BY 4.0 International license.

C) Conservation of a predicted CRE motif (red) in the upstream regulatory sequences of a subset of *pyt-1* orthologs in other nematodes. Sequences were acquired from WormBase ParaSite BLAST (https://parasite.wormbase.org/). Sequences were aligned with Clustal Omega (https://www.ebi.ac.uk/Tools/msa/clustalo/). Formatting was performed with Jalview.

D) Summary of signaling pathways translating temperature stimuli into changes in expression of *dac-1* and *pyt-1*.

Figure S4. DAC-1 does not regulate DAF-7 TGF-β or DAF-28 insulin in ASI or ASJ (Related to Figure 6).

A) (Left) Representative thermotaxis behavior trajectories of adult wild-type and *dac-1(gk211)* animals in a single assay on thermal gradients grown at the indicated conditions. Vertical dashed lines indicate starting temperature on the gradient. (Right) Quantification of thermotaxis assay end points relative to starting position. Each dot indicates the average end point distance of all animals in a single assay. Vertical and horizontal lines indicate mean and SD, respectively. n = 5-8 assays of 15-25 animals each from at least two independent days.

B,C) Quantification of *daf-7*p::GFP (B) and *daf-28*p::GFP (C) in ASI and/or ASJ neurons of L1 larvae grown at 27°C. Each dot is a measurement from a single neuron. Horizontal and vertical lines indicate mean and SD, respectively. n = 28-42 neurons from at least two biologically independent experiments. * indicates different at $p<0.05$ (t test); ns – not significant.

Figure S5. PYT-1 is conserved in a subset of nematodes and contains conserved PY motifs (Related to Figure 7).

bioRxiv preprint doi: https://doi.org/10.1101/2023.01.22.525070; this version posted January 22, 2023. The copyright holder for this preprint (which was not certified by peer review) is the author/funder, who has granted bioRxiv a license to display the preprint in perpetuity. It is made available under aCC-BY 4.0 International license.

A) Phylogenetic tree of PYT-1 and orthologs. PYT-1 orthologs and their sequences were acquired from WormBase ParaSite BLAST (https://parasite.wormbase.org/). Sequences were aligned and the phylogenetic tree was constructed with Clustal Omega (https://www.ebi.ac.uk/Tools/msa/clustalo/). Formatting was performed with Geneious Prime.

B) Conservation of the PPxY and LPxY motifs in the C-terminal domains of PYT-1 and orthologs in other nematodes.

C) (Left) Representative images of endogenously tagged GCY-18::GFP and *gcy-8*p::ARL-13::RFP localization at the AFD sensory endings. The cilium is indicated with an arrowhead. Scale bar: 2 μm. Anterior is at left. (Middle) Quantification of AFD sensory ending morphology in adult animals from the indicated genotypes grown at 15°C overnight and then shifted to 25°C for 4 hours before imaging. AFD sensory endings were visualized with endogenously tagged GCY-18::GFP. n = 39-49 animals. (Right) Quantification of GCY-18::GFP levels in adult animals from the indicated genotypes grown at 15°C overnight and then shifted to 25°C for 4 hours before imaging. Each dot is a measurement from a single AFD sensory ending. Horizontal and vertical lines indicate mean and SD, respectively. n = 23-39 sensory endings from at least two biologically independent experiments. ns – not significant (Fisher's exact test or t test).

Figure S6. CREB-mediated upregulation of PYT-1 is necessary only under specific temporal conditions to regulate neuronal plasticity (Related to Figure 7).

A) Quantification of T^*_{AFD} measured via GCaMP imaging at the AFD sensory endings in wild-type and *pyt-1* animals at the indicated temperature conditions. Each dot is a measurement from a single AFD neuron. Horizontal and vertical bars are the mean and SD, respectively. n = 29-31 animals from at least two biologically independent experiments.

bioRxiv preprint doi: https://doi.org/10.1101/2023.01.22.525070; this version posted January 22, 2023. The copyright holder for this preprint (which was not certified by peer review) is the author/funder, who has granted bioRxiv a license to display the preprint in perpetuity. It is made available under aCC-BY 4.0 International license.

B,C) GCaMP traces from AFD in adult animals during the temperature ramp protocol (green line). Thick lines and shading indicate the average ΔF/F change and SEM, respectively. These traces come from the 15°C or 25°C O/N condition quantified in Figure 7E.

D) Representative trajectories of wild-type and *pyt-1* mutants in a single assay on an isothermal plate at 20°C. Animals were grown under temperature conditions used for positive thermotaxis (see Figure 7G). n = 3 assays of 15-25 animals.

E,F) (Left) GCaMP traces from AFD in adult animals grown at the indicated conditions during the temperature ramp protocol (green line). Thick lines and shading indicate the average ΔF/F change and SEM, respectively. (Right) Quantification of T^*_{AFD} for the indicated genotypes grown at the indicated conditions. Each dot is a measurement from a single animal. Horizontal and vertical lines indicate mean and SD, respectively. n = 13-37 animals from at least two biologically independent experiments.

G) Quantification of T^*_{AFD} from animals of the indicated genotypes grown at the indicated conditions. Each dot is a measurement from a single animal. Horizontal and vertical lines indicate mean and SD, respectively. n = 17-55 animals from at least two biologically independent experiments.

For all panels, ** and *** indicate different at $p<0.01$ and 0.001, respectively (t test,); ns – not significant.

bioRxiv preprint doi: https://doi.org/10.1101/2023.01.22.525070; this version posted January 22, 2023. The copyright holder for this preprint (which was not certified by peer review) is the author/funder, who has granted bioRxiv a license to display the preprint in perpetuity. It is made available under aCC-BY 4.0 International license.

Table S1 related to Figure 2. Temperature-regulated expression changes of examined reporter genes.

Gene	Reporter	Temperature conditions examined	Expression fold-change of reporter in AFD	Expression pattern
gcy-18	*gcy-18*^{crispr}*::gfp (oy165)*	25°C overnight	1.8	AFD sensory endings only
ins-39	*ins-39*^{crispr}*::SL2::gfp (oy167)*	25°C overnight	2.4	AFD only
dac-1	*dac-1*^{crispr}*::gfp (oy172)*	25°C overnight	9.3	AFD, hypodermal cell, neuron
F08H9.4	*F08H9.4*^{crispr}*::gfp (syb5551)*	15°C >25°C 4 hrs	>10	AFD only
pyt-1	*pyt-1*^{crispr}*::gfp (oy169)*	15°C >25°C 4hrs	>10	AFD sensory endings only
F23D12.3	Ex*[F23D12.3*p*::gfp]*	25°C overnight	4.9	AFD only
gcy-29	Ex*[gcy-29*p*::gfp]*	25°C >15°C 4 hrs	9.4	AFD only
droe-4	Ex*[droe-4*p*::gfp]*	25°C overnight, 15°C >25°C 4 hrs	no change	AFD + head neurons
zig-4	Ex*[zig-4*p*::gfp]*	25°C overnight, 15°C >25°C 4 hrs	not expressed in AFD	head neurons
T25B6.4	Ex*[T25B6.4*p*::gfp]*	25°C overnight, 15°C >25°C 4 hrs	not expressed in AFD	head neurons

bioRxiv preprint doi: https://doi.org/10.1101/2023.01.22.525070; this version posted January 22, 2023. The copyright holder for this preprint (which was not certified by peer review) is the author/funder, who has granted bioRxiv a license to display the preprint in perpetuity. It is made available under aCC-BY 4.0 International license.

Table S2. List of strains used in this work.

Strain	Genotype	Source/parent strains	Relevant figures
Wild-type	N2 (Bristol)	CGC	6B-D, 7G, S4A, S6D
PY11298	*oyIs95[ttx-1*p*::egfp::rpl-1a + lin-15(+)]; lin-15(n765ts)*	This paper	1A-D, S1A-C
PHX5551	*F08H9.4(syb5551[F08H9.4::gfp])*	Suny Biotech	2A-C, 6B
PY12306	*pyt-1(oy169[pyt-1::gfp])*	This paper	2A-D, 3A, 3B, 4C, 4F, 5E
PY12307	*dac-1(oy172[dac-1::gfp])*	This paper	2A-C, 3A, 3B, 4B, 4E, 5C, 6A S2D
PY12304	*ins-39(oy167[ins-39::sl2::gfp])*	This paper	2A-C
PY12303	*gcy-18(oy165[gcy-18::gfp])*	This paper	2A-C, S5C
PY12313	Ex*[F23D12.3*p*::gfp + unc-122*p*::tagRfp]*	This paper	S2B
PY12314	Ex*[gcy-29*p*::gfp + unc-122*p*::mCherry]*	This paper	S2C
PY12315	*tax-4(p678); pyt-1(oy169)*	This paper	4C
PY12316	*dac-1(oy172) tax-4(p678)*	This paper	4B
PY12318	*cmk-1(oy21); pyt-1(oy169)*	This paper	4C
PY12319	*dac-1(oy172); cmk-1(oy21)*	This paper	4B
PY12322	*crh-1(tz2); pyt-1(oy169)*	This paper	4F
PY12323	*dac-1(oy172]) crh-1(tz2)*	This paper	4E
PY12326	*crh-1(tz2); F08H9.4(syb5551)*	This paper	4G
PY12311	*dac-1(oy174 oy172)*	This paper	5C
PY12312	*dac-1(oy175 oy172)*	This paper	5C
PY12310	*pyt-1(oy173 oy169)*	This paper	5E, 7H
VC392	*dac-1(gk211)*	CGC	6B-D, S4A
PY12327	*dac-1(gk211)*; Ex*[gcy-8*p*::dac-1a(cDNA) + unc-122*p*::gfp]*	This paper	6B
PY12328	*dac-1(oy172); oyIs94[ttx-1*p*::tagRfp]*	This paper	6A
FK181	*ksIs2[(daf-7*p*::gfp + rol-6(su1006)]*	CGC	S4B
PY12329	*dac-1(gk211); kyIs2[(daf-7*p*::gfp + rol-6(su1006)]*	This paper	S4B
GR1455	*mgIs40[daf-28*p*::nls::gfp + lin-15(+)]*	CGC	S4C
PY12330	*dac-1(gk211); mgIs40[daf-28*p*::nls::gfp + lin-15(+)]*	GR1455; This paper	S4C
PY12331	*pyt-1(oy169);* Ex*[ttx-1*p*::arl-13::tagRfp + unc-122*p*::tagRfp]*	This paper	7C
DCR3055	*wyIs629[gcy-8*p*::GCaMP6s + gcy-8*p*::mCherry + unc-122*p*::gfp]*	Hawk et al., 2018	7D, 7E, 7H, S6A-G

bioRxiv preprint doi: https://doi.org/10.1101/2023.01.22.525070; this version posted January 22, 2023. The copyright holder for this preprint (which was not certified by peer review) is the author/funder, who has granted bioRxiv a license to display the preprint in perpetuity. It is made available under aCC-BY 4.0 International license.

PY12332	*pyt-1(oy160); wyIs629[gcy-8*p*::GCaMP6s + gcy-8*p*::mCherry + unc-122*p*::gfp]*	This paper	7D, 7E, 7H, S6A-C, S6E, S6F
PY12333	*pyt-1(oy160); wyIs629[gcy-8*p*::GCaMP6s + gcy-8*p*::mCherry + unc-122*p*::gfp]; Ex[gcy-8*p*:: pyt-1(genomic)::sl2::mCherry + unc-122*p*::tagRfp]* Line 1	This paper	7E
PY12334	*pyt-1(oy160); wyIs629[gcy-8*p*::GCaMP6s + gcy-8*p*::mCherry + unc-122*p*::gfp]; Ex[gcy-8*p*:: pyt-1(genomic)::sl2::mCherry + unc-122*p*::tagRfp]* Line 2	This paper	7E
PY12101	*oyIs96[gcy-8*p*::FlincG3 + gcy-8*p*::myrtagRfp + unc-122*p*::dsRed]*	This paper	7F
PY12335	*pyt-1(oy160); oyIs96[gcy-8*p*::FlincG3 + gcy-8*p*::myrtagRfp + unc-122p::dsRed]*	This paper	7F
PY12302	*pyt-1(oy160)*	This paper	7G, S6D
PY12336	*crh-1(tz2); wyIs629[gcy-8*p*::GCaMP6s + gcy-8*p*::mCherry + unc-122*p*::gfp]*	This paper	S6G
PY12337	*pyt-1(oy169); wyIs629[gcy-8*p*::GCaMP6s + gcy-8*p*::mCherry + unc-122*p*::gfp]*	This paper	7H, S6E, S6F
PY12338	*pyt-1(oy173 oy169); wyIs629[gcy-8*p*::GCaMP6s + gcy-8*p*::mCherry + unc-122*p*::gfp]*	This paper	7H, S6E, S6F
PY12339	*Ex[dac-1b*p*::gfp + unc-122::tagRfp]*	This paper	S3A, S3B
PY12340	*gcy-18(oy165);* Ex*[ttx-1p::arl-13::tagRfp + unc-122::tagRfp]*	This paper	S5C
PY12341	*gcy-18(oy165); pyt-1(oy160);* Ex*[ttx-1*p*::arl-13::tagRfp + unc-122*p*::tagRfp]*	This paper	S5C
PY12342	Ex*[droe-4*p*::gfp + ttx-1*p*::tagRfp + unc-122*p*::tagRfp]* Line 1	This paper	Table S1
PY12343	Ex*[droe-4*p*::gfp + ttx-1*p*::tagRfp + unc-122*p*::tagRfp]* Line 2	This paper	Table S1
PY12344	Ex*[T25B6.4*p*::gfp + ttx-1*p*::tagRfp + unc-122*p*::tagRfp]* Line 1	This paper	Table S1
PY12345	Ex*[T25B6.4*p*::gfp + ttx-1*p*::tagRfp + unc-122*p*::tagRfp]* Line 2	This paper	Table S1
PY12346	Ex*[zig-4*p*::gfp + ttx-1*p*::tagRfp + unc-122*p*::tagRfp]* Line 1	This paper	Table S1
PY12347	Ex*[zig-4*p*::gfp + ttx-1*p*::tagRfp + unc-122*p*::tagRfp]* Line 2	This paper	Table S1

bioRxiv preprint doi: https://doi.org/10.1101/2023.01.22.525070; this version posted January 22, 2023. The copyright holder for this preprint (which was not certified by peer review) is the author/funder, who has granted bioRxiv a license to display the preprint in perpetuity. It is made available under aCC-BY 4.0 International license.

Table S3. List of plasmids used in this work.

**Plasmid name**	**Description**	**Promoter length**	**Source**
PSAB1292	*ttx-1*p*::egfp::rpl-1a*	2.6 kb	This paper
PSAB1304	*F23D12.3*p*::gfp*	1 kb	This paper
PSAB1305	*gcy-29*p*::gfp*	1.8 kb	This paper
PSAB1306	*ttx-1*p*::tagRfp*	800 bp	This paper
PSAB1307	*droe-4*p*::gfp*	1.7 kb	This paper
PSAB1308	*T25B6.4*p*::gfp*	3 kb	This paper
PSAB1309	*zig-4*p*::gfp*	2.8 kb	This paper
PSAB1310	*gcy-8*p*:dac-1a:SL2:mCherry*	645 bp	This paper
PSAB1315	*gcy-8*p*::myrtagRfp*	776 bp	Gift from Ashish K. Maurya
PSAB1311	*ttx-1*p*::arl-13::tagRfp*	800 bp	This paper
PSAB1312	*gcy-8*p*::pyt-1::SL2::mCherry*	645 bp	This paper
pFG248	*gcy-8*p*::FlincG3*	2.2 kb	Woldemariam et al., 2019
PSAB1313	*dac-1a*p*:gfp*	2.4 kb	This paper
PSAB1314	*dac-1b*p*:gfp*	447 bp	This paper

bioRxiv preprint doi: https://doi.org/10.1101/2023.01.22.525070; this version posted January 22, 2023. The copyright holder for this preprint (which was not certified by peer review) is the author/funder, who has granted bioRxiv a license to display the preprint in perpetuity. It is made available under aCC-BY 4.0 International license.

REFERENCES

Hawk, J.D., Calvo, A.C., Liu, P., Almoril-Porras, A., Aljobeh, A., Torruella-Suárez, M.L., Ren, I., Cook, N., Greenwood, J., Luo, L., et al. (2018). Integration of Plasticity Mechanisms within a Single Sensory Neuron of C. elegans Actuates a Memory. Neuron *97*, 356-367.e4. 10.1016/j.neuron.2017.12.027.

Hobert, O. (2013). The neuronal genome of Caenorhabditis elegans. WormBook, 1–106. 10.1895/wormbook.1.161.1.

Valperga, G., and de Bono, M. (2022). Impairing one sensory modality enhances another by reconfiguring peptidergic signalling in Caenorhabditis elegans. Elife *11*, e68040. 10.7554/eLife.68040.

Woldemariam, S., Nagpal, J., Hill, T., Li, J., Schneider, M.W., Shankar, R., Futey, M., Varshney, A., Ali, N., Mitchell, J., et al. (2019). Using a Robust and Sensitive GFP-Based cGMP Sensor for Real-Time Imaging in Intact Caenorhabditis elegans. Genetics *213*, 59–77. 10.1534/genetics.119.302392.

bioRxiv preprint doi: https://doi.org/10.1101/2023.01.22.525070; this version posted January 22, 2023. The copyright holder for this preprint (which was not certified by peer review) is the author/funder, who has granted bioRxiv a license to display the preprint in perpetuity. It is made available under aCC-BY 4.0 International license.

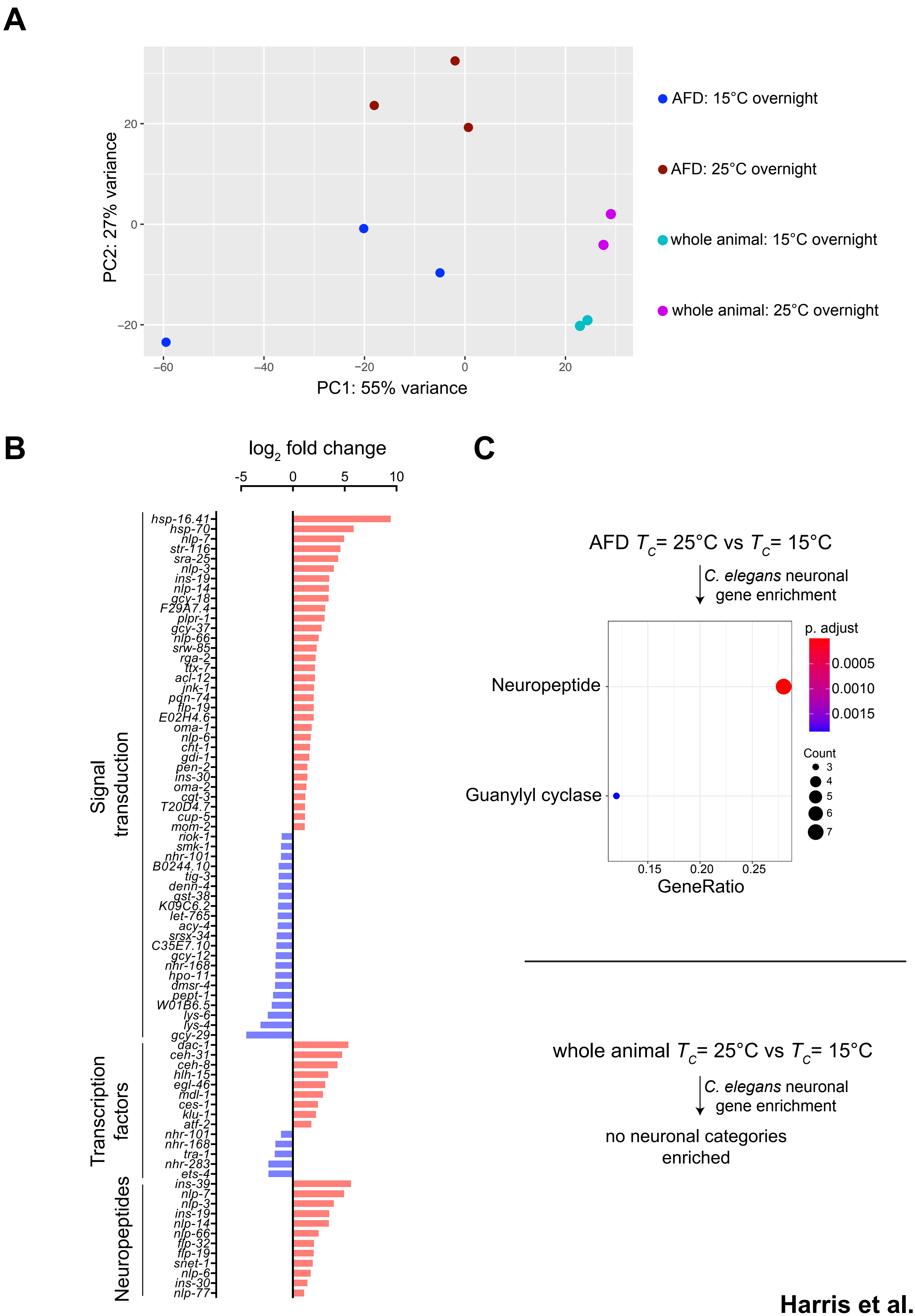

Harris et al., Fig. S1

bioRxiv preprint doi: https://doi.org/10.1101/2023.01.22.525070; this version posted January 22, 2023. The copyright holder for this preprint (which was not certified by peer review) is the author/funder, who has granted bioRxiv a license to display the preprint in perpetuity. It is made available under aCC-BY 4.0 International license.

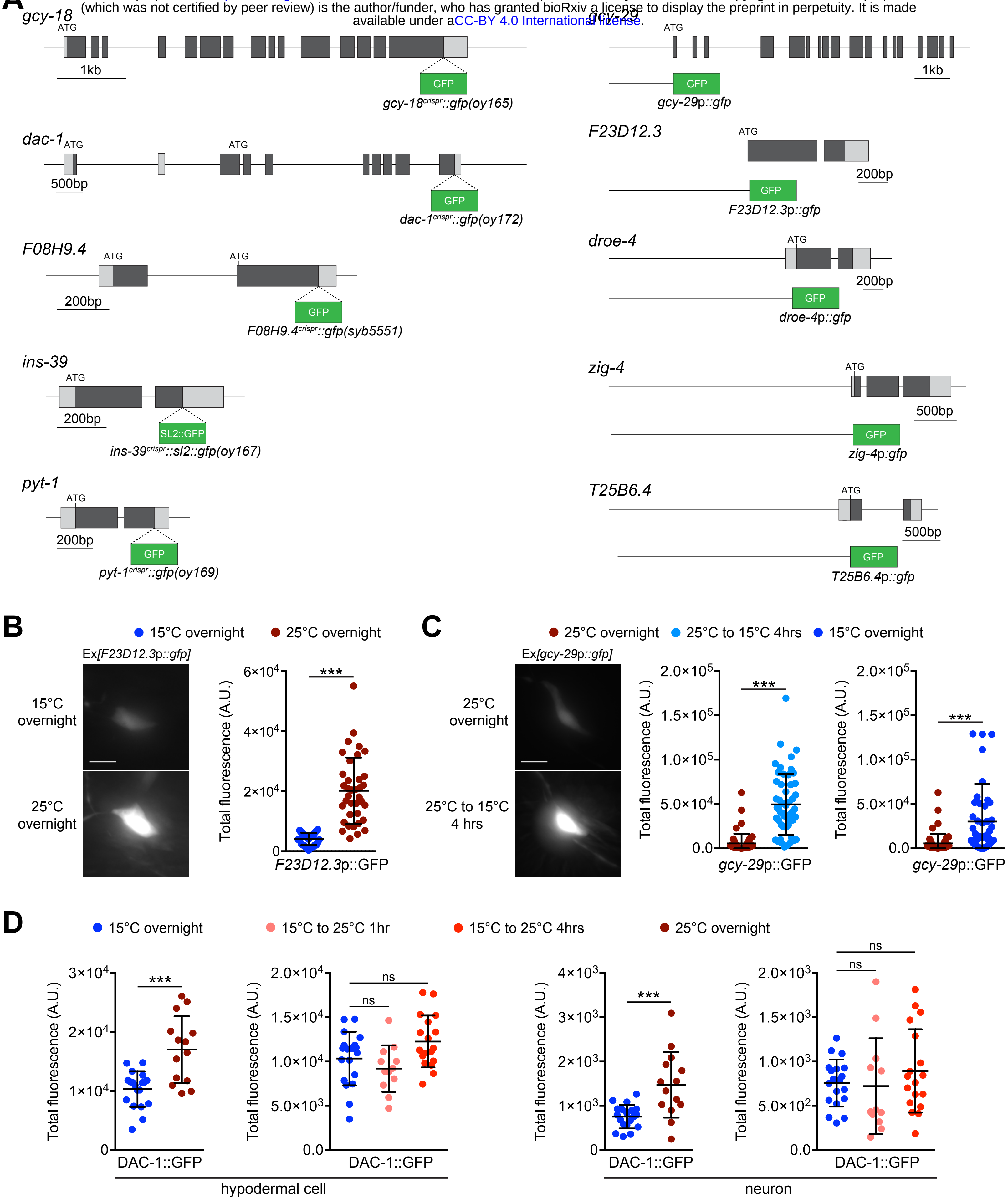

Harris et al., Fig. S2

bioRxiv preprint doi: https://doi.org/10.1101/2023.01.22.525070; this version posted January 22, 2023. The copyright holder for this preprint (which was not certified by peer review) is the author/funder, who has granted bioRxiv a license to display the preprint in perpetuity. It is made available under aCC-BY 4.0 International license.

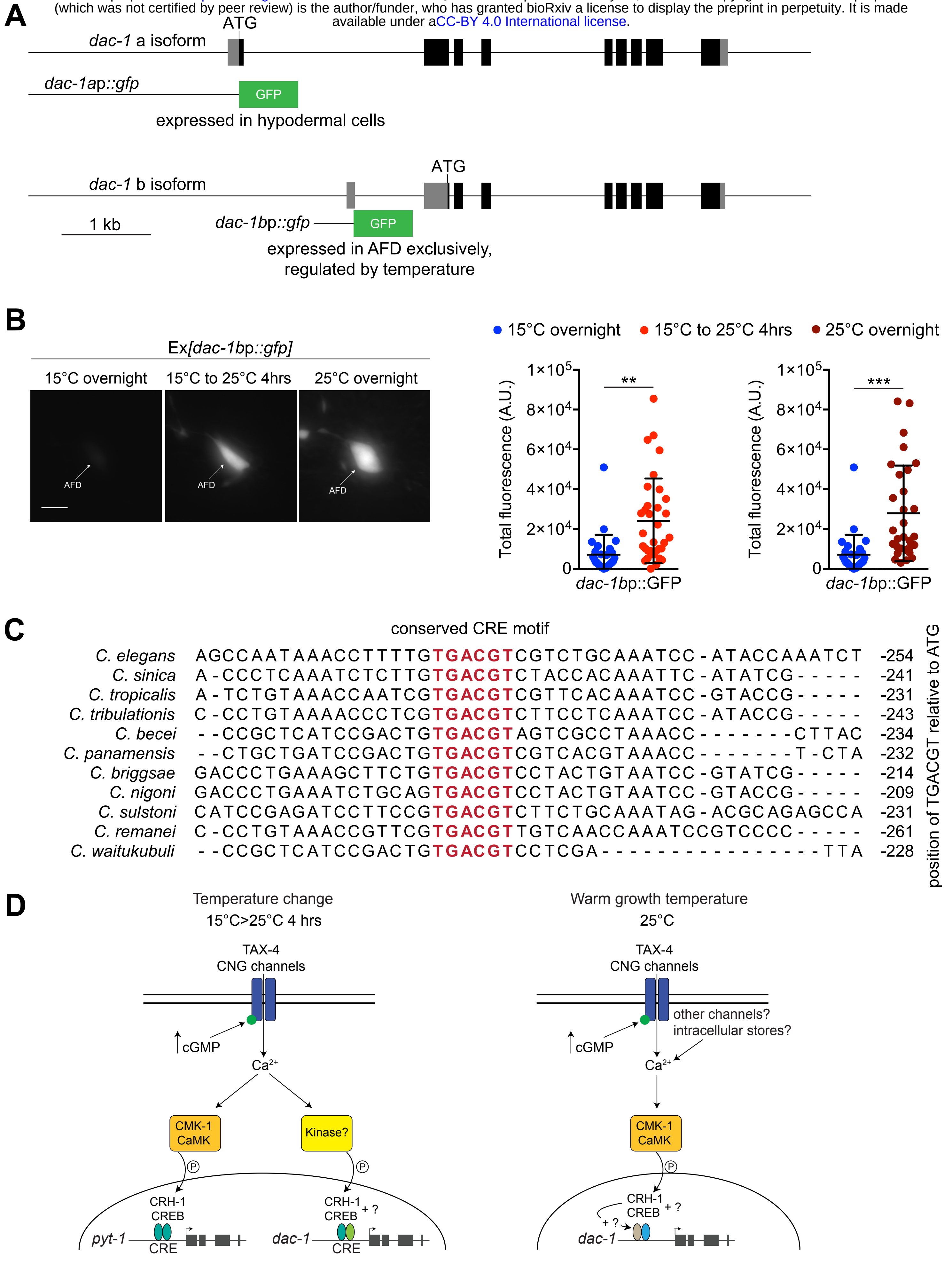

Harris et al., Fig. S3

bioRxiv preprint doi: https://doi.org/10.1101/2023.01.22.525070; this version posted January 22, 2023. The copyright holder for this preprint (which was not certified by peer review) is the author/funder, who has granted bioRxiv a license to display the preprint in perpetuity. It is made available under aCC-BY 4.0 International license.

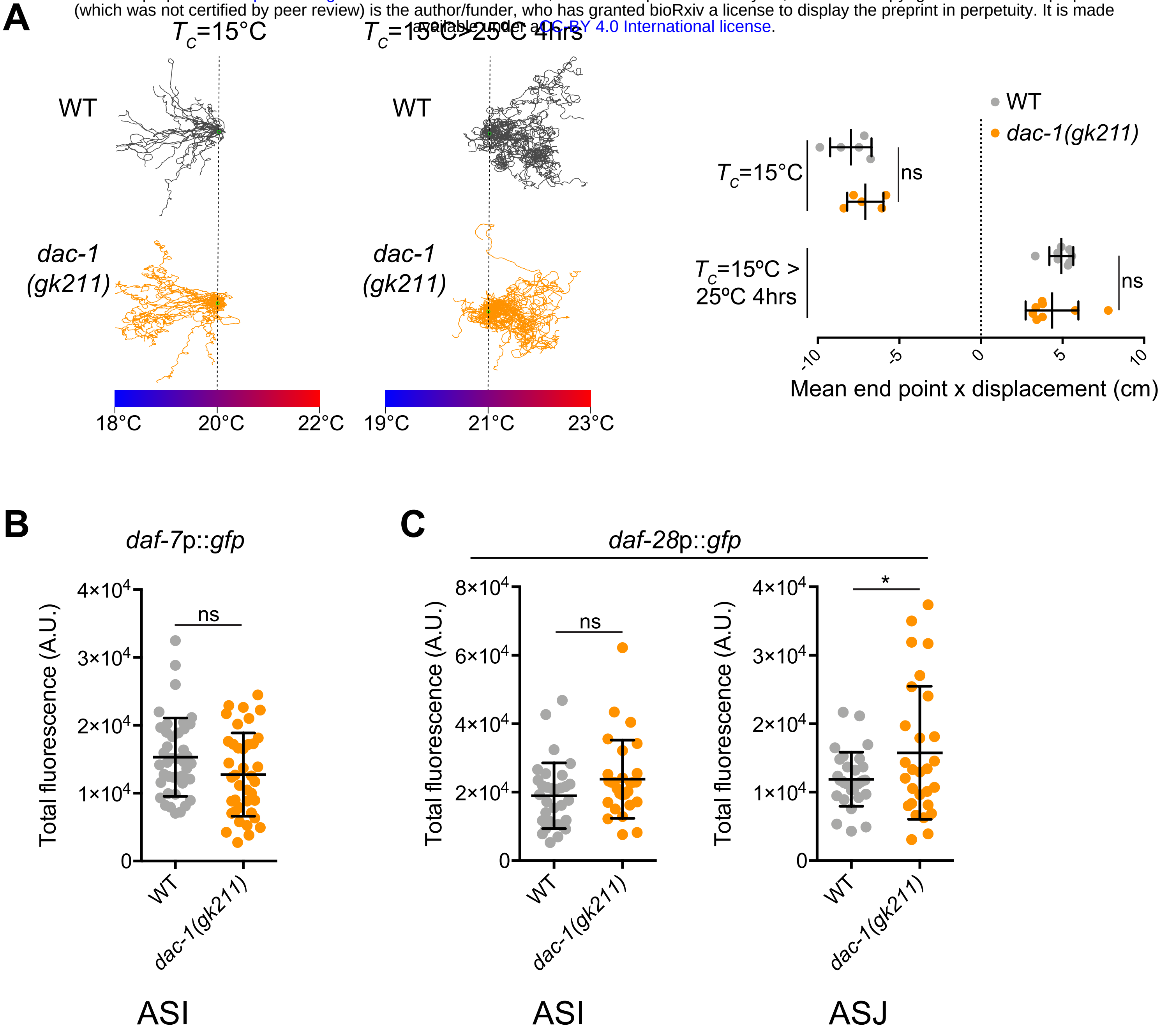

Harris et al., Fig. S4

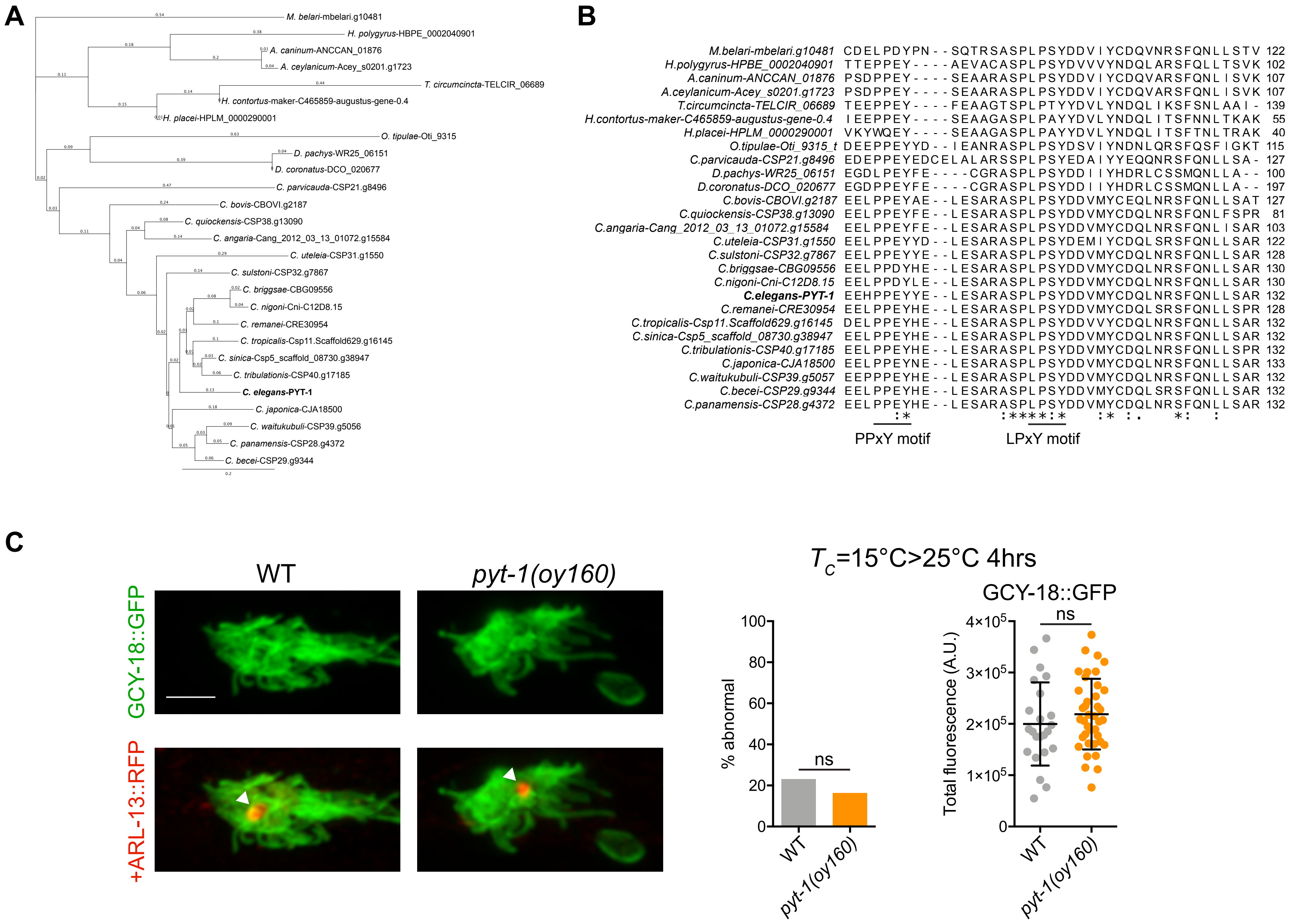

Harris et al., Fig. S5

bioRxiv preprint doi: https://doi.org/10.1101/2023.01.22.525070; this version posted January 22, 2023. The copyright holder for this preprint (which was not certified by peer review) is the author/funder, who has granted bioRxiv a license to display the preprint in perpetuity. It is made available under aCC-BY 4.0 International license.

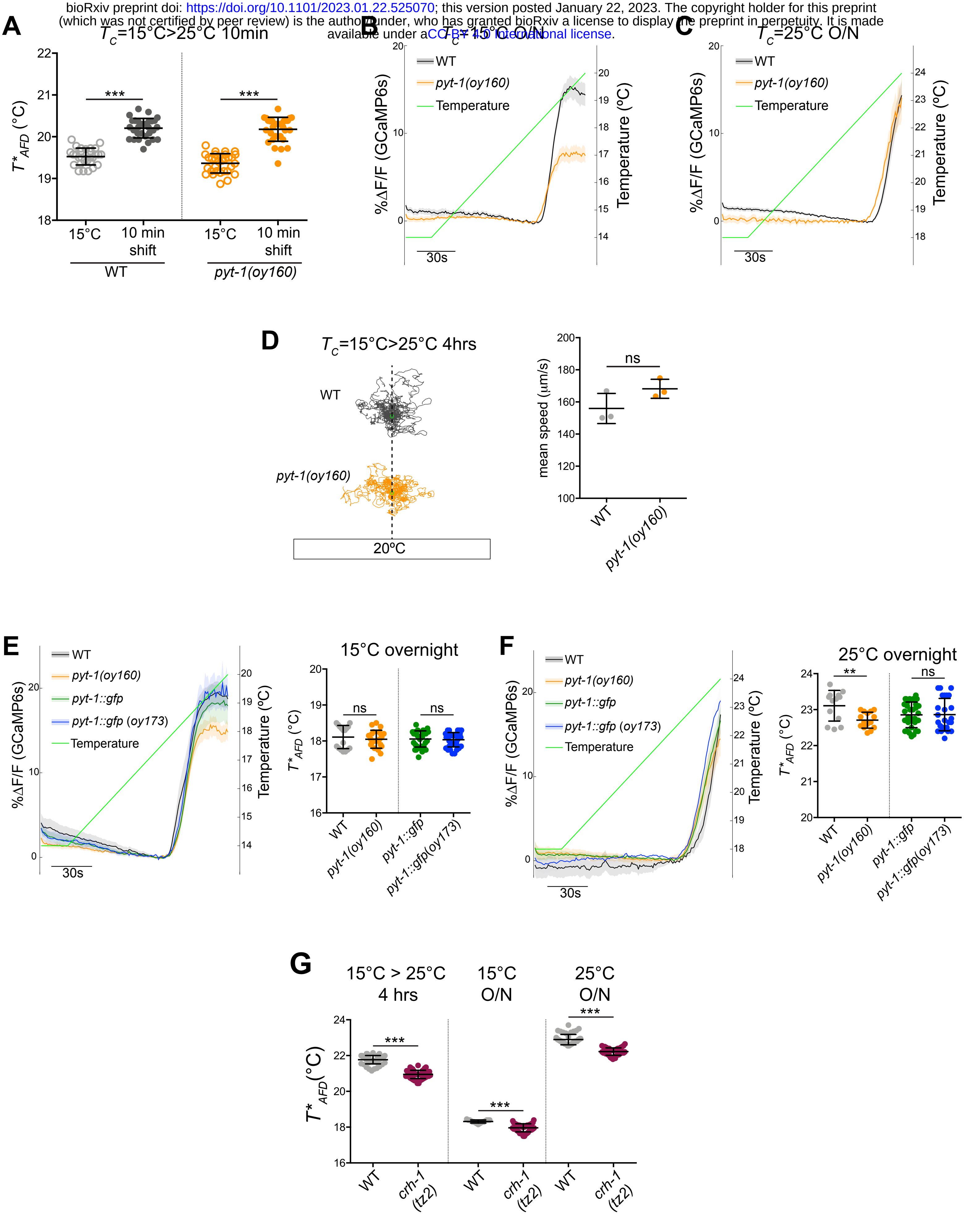

Harris et al., Fig. S6
